# Antarctic marine ciliates under stress: superoxide dismutases from the psychrophilic *Euplotes focardii* are cold-active yet heat tolerant enzymes

**DOI:** 10.1038/s41598-018-33127-1

**Published:** 2018-10-03

**Authors:** Alessandro Pischedda, Kesava Priyan Ramasamy, Marco Mangiagalli, Federica Chiappori, Luciano Milanesi, Cristina Miceli, Sandra Pucciarelli, Marina Lotti

**Affiliations:** 1 Department of Biotechnology and Biosciences, University of Milano-Bicocca, Piazza della Scienza 2, 20126 Milano, Italy; 2 School of Biosciences and Veterinary Medicine, University of Camerino, Via Gentile III da Varano, 1, 62032 Camerino (MC), Italy; 3 Institute of Biomedical Technologies – CNR, Segrate (MI), Italy

## Abstract

Oxidative stress is a particularly severe threat to Antarctic marine polar organisms because they are exposed to high dissolved oxygen and to intense UV radiation. This paper reports the features of three superoxide dismutases from the Antarctic psychrophilic ciliate *Euplotes focardii* that faces two environmental challenges, oxidative stress and low temperature. Two out of these are Cu,Zn superoxide dismutases (named *Ef*-SOD1a and *Ef*-SOD1b) and one belongs to the Mn-containing group (*Ef*-SOD2). *Ef*-SOD1s and *Ef*-SOD2 differ in their evolutionary history, expression and overall structural features. *Ef*-SOD1 genes are expressed at different levels, with *Ef*-SOD1b mRNA 20-fold higher at the ciliate optimal temperature of growth (4 °C). All *Ef*-SOD enzymes are active at 4 °C, consistent with the definition of cold-adapted enzymes. At the same time, they display temperatures of melting in the range 50–70 °C and retain residual activity after incubation at 65–75 °C. Supported by data of molecular dynamics simulation, we conclude that the *E. focardii* SODs combine cold activity, local molecular flexibility and thermo tolerance.

## Introduction

Polar marine organisms face a number of environmental challenges, in particular the adverse effects of cold on key biological processes and high oxidative stress^1,2^. Low temperature greatly impairs enzyme activity and membrane fluidity, slows down secretory processes and affects the stability and activity of macromolecular machines (replication and transcription complexes, mitotic spindles, ribosomes) which depend on weak, noncovalent molecular interactions^3–5^. To survive, “psychrophilic” organisms rely on adaptive changes, that include the up-regulation of genes encoding proteins involved in metabolite transport, the synthesis of cryoprotectors (mannitol, polyamines), increased membrane fluidity and the production of enzymes endowed with high activity at low temperature^4^. Moreover, cold-adapted marine organisms from Antarctica are exposed to both high concentration of oxygen and to UV radiation (the latter due to the ozone depletion) that boost the production of reactive oxygen species (ROS). ROS are involved in several physiological processes, such as cellular signaling pathways, resistance to microbial pathogens, apoptosis^6^. Nevertheless, at high concentration ROS threaten essential cellular macromolecules, notably proteins, lipids, and nucleic acids. As a consequence, antioxidant defences are of key relevance to aquatic polar living beings, as ROS control is required to balance their concentration and avoid cellular damage^7^. One of the key players in this process are the ubiquitous metalloenzymes superoxide dismutases (SOD, EC 1.15.1.1) that catalyze the dismutation of superoxide anions into molecular oxygen and hydrogen peroxide $({{{\rm{O}}}_{{\rm{2}}}}^{-}+{{{\rm{O}}}_{{\rm{2}}}}^{-}+{{\rm{2H}}}^{+}\to {{\rm{O}}}_{{\rm{2}}}+{{\rm{H}}}_{{\rm{2}}}{{\rm{O}}}_{{\rm{2}}})$^8,9^.

SODs are grouped into three protein families, based on the metal cofactor they contain and on the protein fold^10^. Copper,zinc SODs (Cu,Zn SODs) are found in the cytoplasm of eukaryotes, in the chloroplasts of some plants and in the periplasmic space of bacteria^11,12^. This group of SODs is often referred to as SOD1. SOD1s are homodimers, with each subunit organized in eight antiparallel β strands and three external loops^13^. SOD3s, the Cu,Zn enzymes present in the extracellular fluids of eukaryotes, are similar to the previous ones but have tetrameric quaternary structure^14,15^. Iron- and manganese-containing SODs (FeSOD and MnSOD) are believed to be the more primitive forms of SODs^16,17^. FeSODs are found in prokaryotes and chloroplasts, while MnSODs are present both in prokaryotes and in the mitochondrial matrix of eukaryotes. Several fungi possess both cytosolic and mitochondrial MnSODs^18^. Cambialistic SODs, whose activity depends either on iron or on manganese incorporated in the same protein moiety, have been identified in a few classes of bacteria and eukaryotes^19–21^. MnSOD and FeSOD can be either homodimers or homotetramers and share high similarity in sequence and structure^22,23,24^, strongly suggesting a common evolutionary origin. In contrast, Cu,Zn and Mn/Fe SODs never shared a common ancestry, as shown by their distinctive amino acid sequences^25^ and completely different tertiary structures^13^. The discovery of a nickel containing enzyme in *Streptomyces* and cyanobacteria established a novel SOD group with a unique Ni-hook structural motif ^26^.

This study focuses on one MnSOD and two Cu,Zn SODs, identified in the strict psychropilic protist *Euplotes focardii*, a hypotrichous ciliated protozoan isolated from the coastal seawaters of Terra Nova Bay in Antarctica^27^. Under laboratory condition, *E. focardii* has an optimal temperature of growth of about 4–5 °C and is not viable above 12 °C^28^. We show that the two SOD families likely followed different evolutionary history. However, they share similar temperature-dependent regulation of expression and combine cold activity with thermo-tolerance, a feature that could have played a role in the successful colonization of the Antarctic marine habitat by this ciliate.

## Results

### Evolution of *E. focardii* superoxide dismutases

Putative Cu,Zn SOD coding sequences identified by BLAST search in the *E. focardii* transcriptome were reported in a previous work and named *Ef*-SOD1a (accession number KF740481) and *Ef*-SOD1b (accession number KF740482)^29^. *Ef*-SOD2 (MnSOD, accession number MG575644) is described in this work.

**Figure 1:**
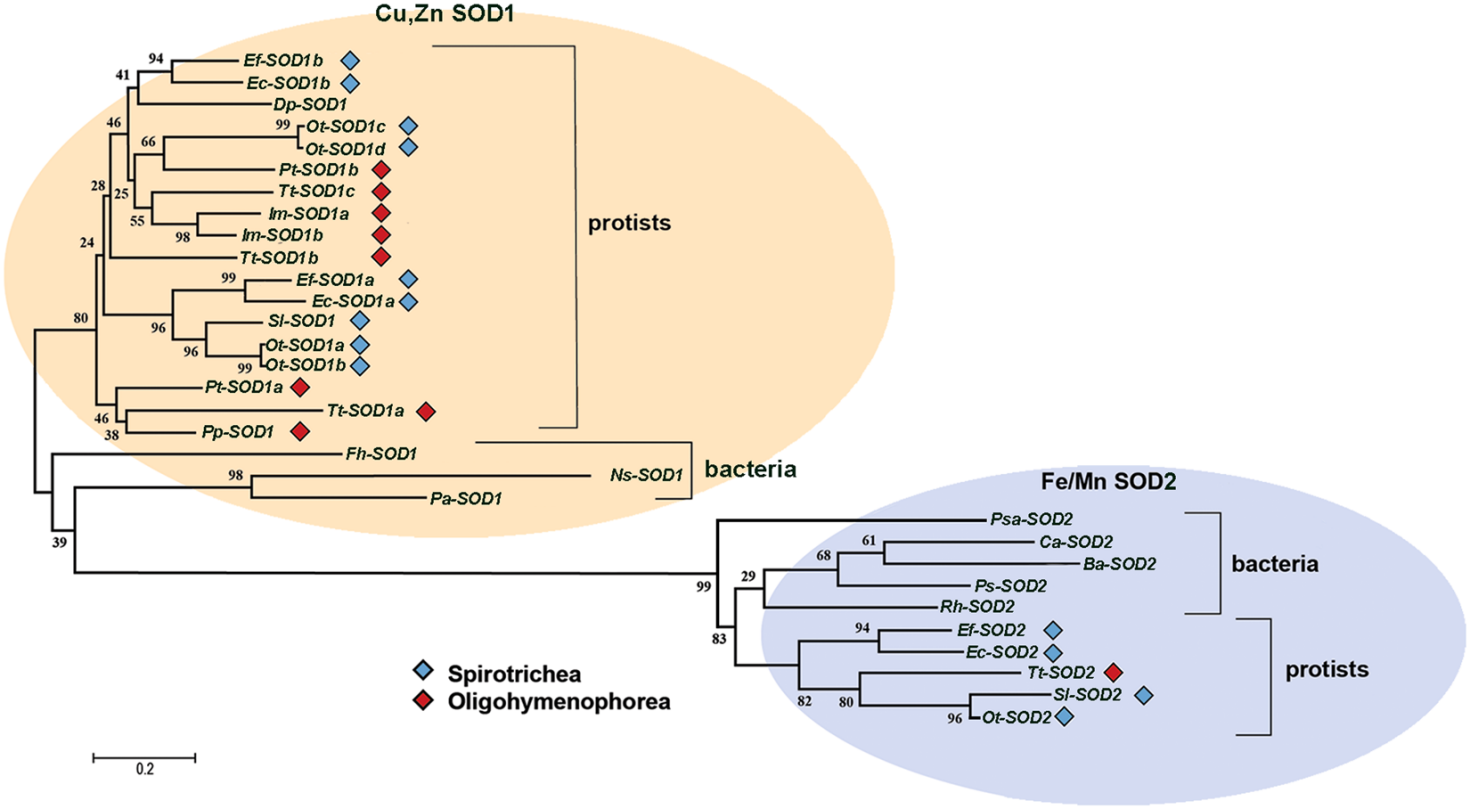
Phylogenetic analysis of *E. focardii* SODs. Cu,Zn (shaded in orange) and Fe/MnSODs (shaded in light blue) from *E. focardii* and other ciliates were compared with the homologous sequences from the protist *Dictyostelium discoideum* and from psychrophilic bacteria. Sources and accession number of SOD enzymes are reported in Table S1. Cyan and red diamond symbols indicate ciliates that belong to Spirotrichea and Oligohymenophorea classes. The tree was obtained by using Neighbour*-*Joining (NJ); the numbers on the branches represent bootstrap values for 1000 replicates.

The molecular evolution of *E. focardii* SODs (*Ef*-SODs) was studied in the frame of known SOD sequences from other species of ciliates. The data set included also the Cu,Zn SOD from the amoeba *Dictyostelium* (*Dp*-SOD1) as a non-ciliate member of the protista group and sequences (both Cu,Zn and Fe/Mn SODs) from Antarctic bacteria to have a reference describing cold-active SODs (Fig. 1). In the phylogenetic tree, SODs assemble in two separate clusters. The two clusters correspond to the different families, SOD1 and SOD2, defined based on the metal cofactors they contain (either Cu,Zn or Fe/Mn). Overall, the length of the branches is consistent with the low degree of sequence conservation highlighted also in the multiple sequence alignments (Figs S1 and S2). Within the Cu,Zn SOD (SOD1) family, *Ef*-SOD1a and *Ef*-SOD1b isoforms cluster in two different clades. The clade that includes *Ef*-SOD1a contains SOD1 sequences from *Euplotes* (*Ec*-SOD1a), *Stylonychia* (*Sl-*SOD1) and *Oxytricha* (*Ot-*SOD1a and *Ot*-SOD1b) all of them belonging to the same class of ciliates (Spirotrichea). By contrast, the clade containing *Ef*-SOD1b is more heterogeneous, since it groups SOD sequences from both Spirotrichea and Oligohymenophorea (*Paramecium*, *Tetrahymena* and *Ichthyophthirius)* and that from *Dictyostelium*. Cu,Zn SODs from Antarctic bacteria (*Flavobacterium hibernum*, *Nesterenkonia* sp.AN1 and *Planococcus antarcticus*) are completely separated from the protistan group. Their position in the tree excludes a close phylogenetic relationship with the homologous sequences from the Antarctic ciliate *E. focardii*. By contrast, the bacterial and the protistan SODs of the Fe/Mn family (SOD2) cluster together in a single clade. In conclusion, the topology of the tree suggests that SOD1 evolutionary history would have witnessed multiple events of gene duplication followed by sequence diversification even inside homogeneous groups of ciliate and bacteria, whereas SOD2 sequences are more conserved.

### Expression of *E. focardii* superoxide dismutases

**Figure 2:**
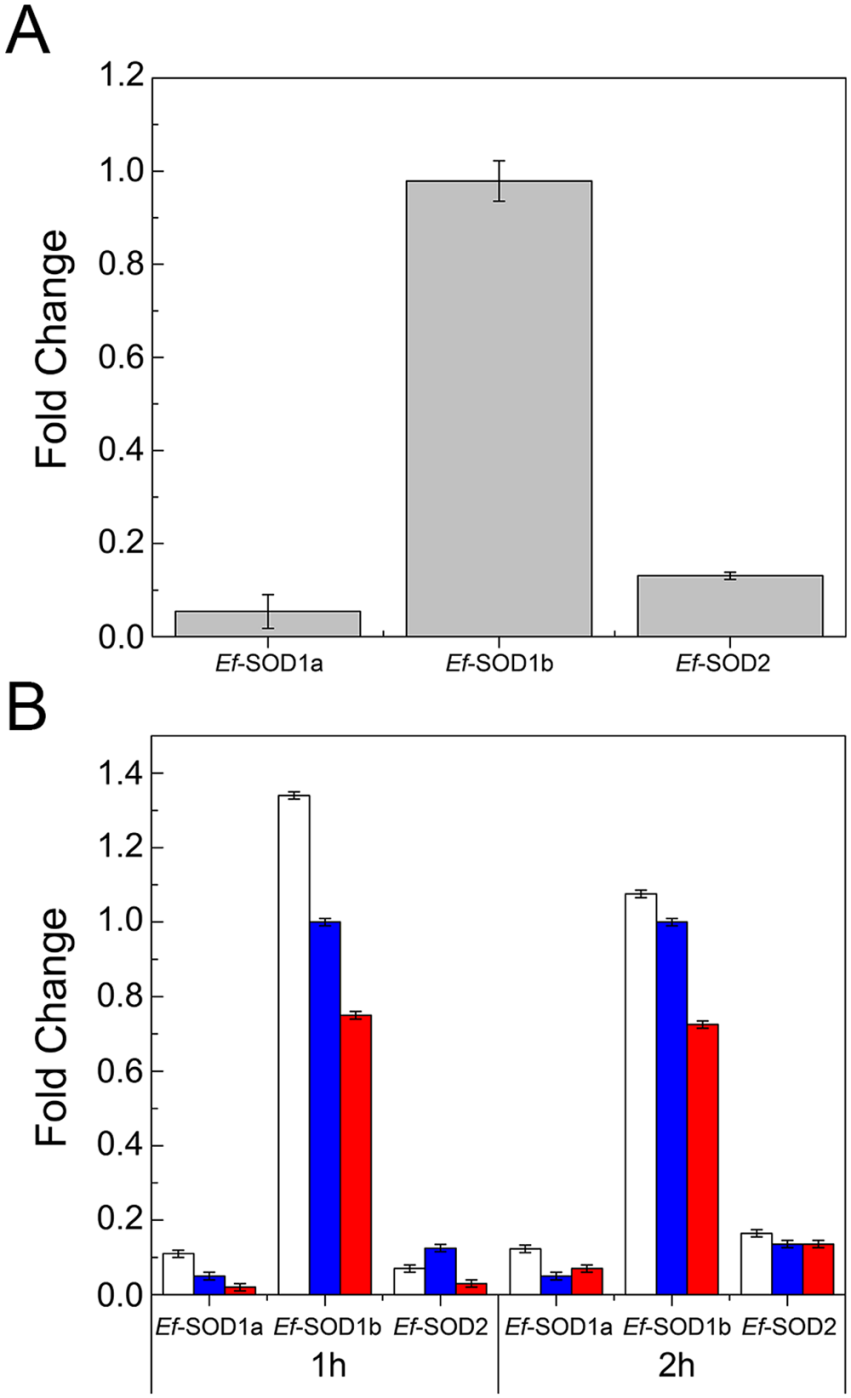
Expression levels of *Ef*-SODs in *E. focardii* cells. SOD mRNAs were quantified by qRT-PCR under growing laboratory conditions (4 °C) (**A**) and compared with values obtained under thermal stress (**B**) applied by incubating *E. focardii* cells at 0 °C (white bars) or 12 °C (red bars). Blue bars are the control levels detected at 4 °C. Results are reported as mean of three independent experiments. Errors bar represent standard deviation. Statistical differences (p-values) between the thermal stress conditions and the controls are all <0.05 (not reported).

In order to investigate how gene expression is regulated by temperature, we analyzed by qRT-PCR the transcription levels of SODs coding genes in *E. focardii* cells both at physiological temperature (4 °C) and under temperature stress that is after exposure either at 0 °C, a mild temperature drop, or at 12 °C, the highest temperature permissive for *E. focardii* growing. At the constant temperature of 4 °C, the three SOD genes are expressed at different levels, with the *Ef-*SOD1b mRNA nearly 20-fold higher and the *Ef*-SOD2 mRNA ca. 2,5-fold higher than that of *Ef*-SOD1a (Fig. 2A). After 1-hour shift at 0 °C both SOD1s were up-regulated (white bars in Fig. 2B), whereas at 12 °C a down-regulation was detected (red bars). *Ef*-SOD2 mRNA levels decreased both at 0 °C and at 12 °C (Fig. 2B). Regulation effects of all *Ef-SODs* fainted with time, hinting to cell adaptation to the new temperature condition (see results at 2 hours of incubation).

### Biochemical and conformational features of recombinant superoxide dismutases

A putative signal peptide of 18 amino acids was identified by PROTTER ver.1.0^30^ at the N-terminus of *Ef*-SOD1a. *In silico* analysis of the 224 amino acids of *Ef*-SOD2 putative protein by MitoFates^31^ identified at its N-terminus a 19 amino acid signal peptide for translocation to mitochondria. Based on these results, in the synthetic genes the corresponding nucleotide stretches were omitted (Fig. S3).

Truncated *Ef*-SOD1a and *Ef*-SOD2 were produced in *E. coli* as soluble proteins and in the following are referred to as *Ef*-SOD2^Δ^ and *Ef-*SOD1a^Δ^. Consistent with the lack of any predicted sequence for transport, the full-length *Ef*-SOD1b sequence was obtained at high yield. Recombinant proteins were produced by 16 hours cultures at 25 °C in Zym5052 medium in the presence or in the absence of Cu and Zn (see materials and methods) for *Ef-*SOD1a^Δ^ and *Ef*-SOD1b, with or without Mn in the case of *Ef*-SOD2^Δ^. Added metals did not produce any effect neither on cell viability nor on the amount of recombinant proteins (data not shown). Protein yields as determined after IMAC purification were 5.3 mg/L *Ef*-SOD2^Δ^, 3.1 mg/L *Ef-*SOD1a^Δ^, 39 mg/L *Ef*-SOD1b.

**Figure 3:**
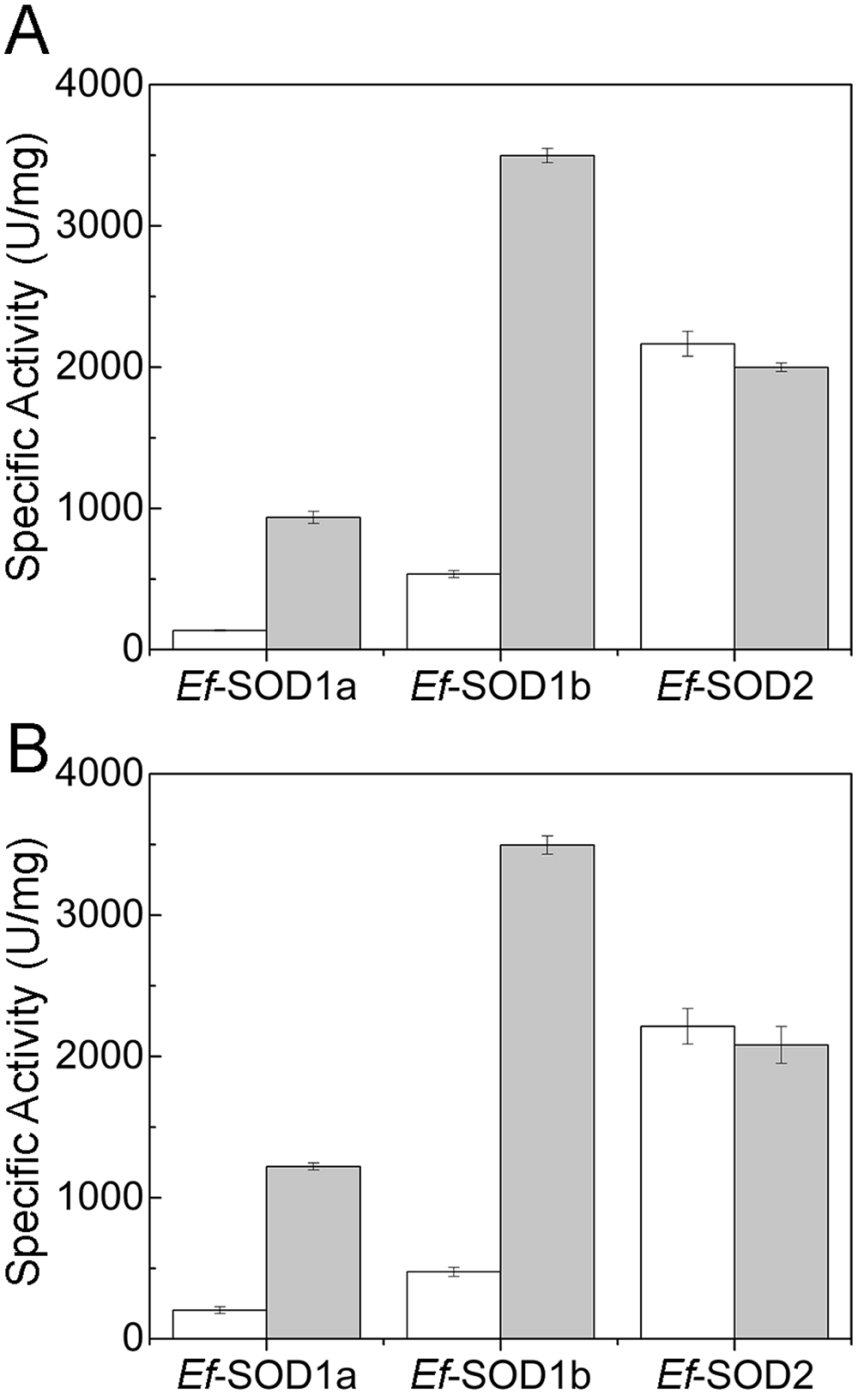
Specific activity of *Ef-*SODs measured at 4 °C (**A**) and 27 °C (**B**). Specific activity of recombinant *Ef*-SODs was measured both in the absence (white) and in the presence (grey) of the respective metal cofactors (Cu and Zn for *Ef*-SOD1s and Mn for *Ef*-SOD2) added at the concentration of 250 µM in the culture medium during the production of recombinant proteins.

We assayed for activity at 4 °C and 27 °C recombinant proteins produced both in the presence and in the absence of metal cofactors in the growth medium during the heterologous expression in *E. coli* (Fig. 3). Given assay temperatures were selected since the first one (4 °C) is the optimal temperature of growth of *E. focardii* cells under laboratory conditions and the second one approaches the optimal temperature of most cold-active enzymes, included a phospholipase and an alpha-amylase previously characterized from this Antarctic ciliate^32,33^.

Overall, the activity of *Ef-*SOD2^Δ^ was poorly affected both by temperature and by added metals. The combined effects of temperature and metals on SOD1s are complex and are therefore described separately. We observed that the specific activities of *Ef-*SOD1a^Δ^ and *Ef*-SOD1b increased by ~6–7 folds in the presence of metals available to cells during production. As for temperature dependence, *Ef*-SOD1a is more active (~25%) at the highest temperature, while *Ef*-SOD1b shows similar activity at 4 °C and 27 °C. Overall, the data presented demonstrate cold activity for all *Ef*-SODs.

**Figure 4:**
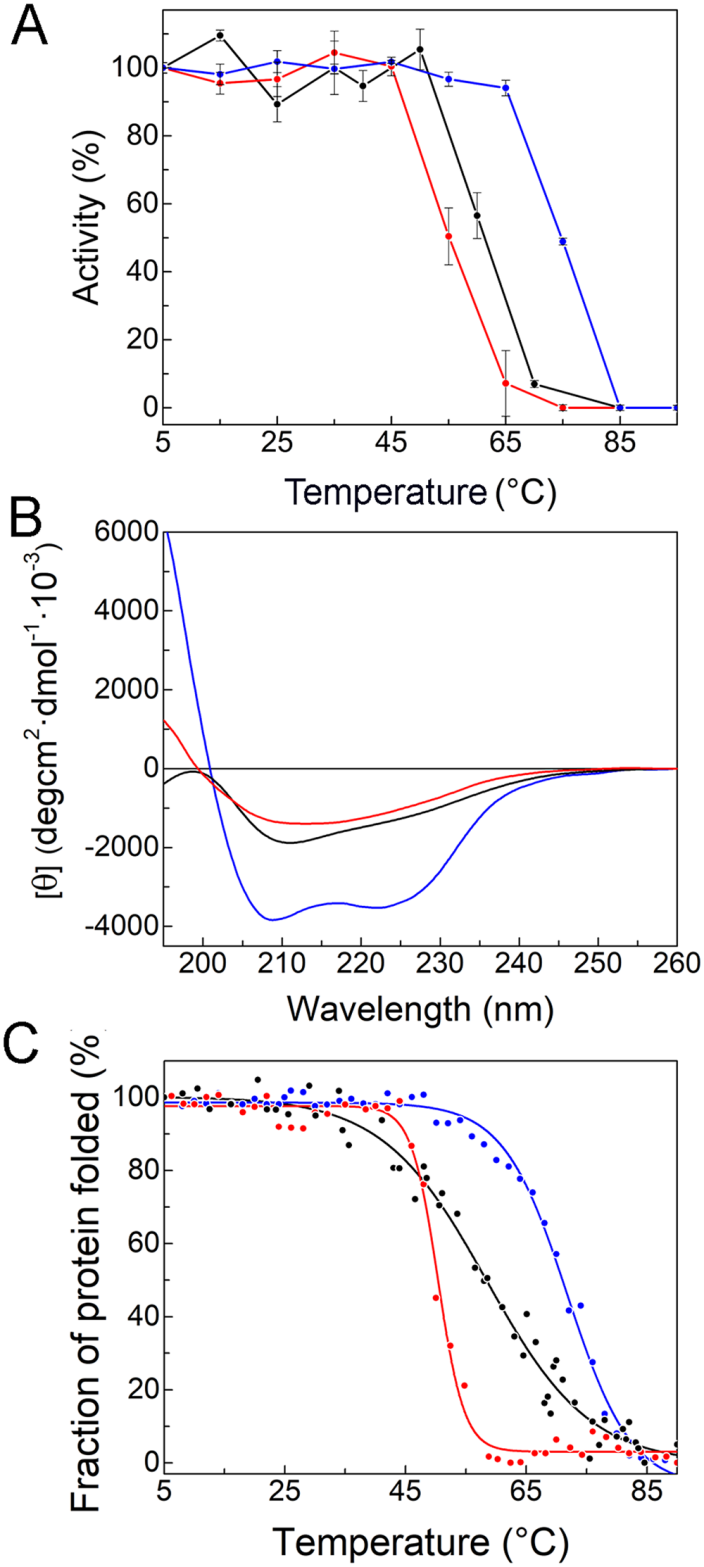
Effect of temperature on *Ef-*SODs activity and structure. (**A**) Samples of *Ef*-SOD1a^Δ^ (black), *Ef*-SOD1b (red), *Ef*-SOD2^Δ^ (blue) were incubated for 20 minutes at indicated temperatures. After incubation, residual activities were assayed at appropriate dilution at room temperature. Measured activity was normalized taking the initial activity as 100%. (**B**) Far UV CD spectra of SODs at 4 °C. (**C**) Thermal stability of *Ef*-SODs. Ellipticity values were recorded at 215 nm (*Ef*-SOD1a^Δ^), 210 nm (*Ef*-SOD1b), 208 nm (*Ef*-SOD2^Δ^) during heating from 5 °C to 90 °C. Initial CD signal was taken as 100% for normalization. Recombinant proteins were produced in the presence of metal cofactors.

In order to gain a deeper insight into the temperature dependence and stability of the three SODs, we measured the residual activity of enzymes pre-incubated 20 minutes at temperatures in the range 5 °C to 90 °C (Fig. 4A). We observed that both *Ef*-SOD1s were kinetically stable in the range 5 °C– 55/60 °C, with midpoints at ~60 °C for *Ef-*SOD1a^Δ^ and of 55 °C for *Ef*-SOD1b, whereas the activity of *Ef*-SOD2^Δ^ kept constant from 5 °C to 70 °C. Pre-incubation above 70 °C induced a sharp decrease in *Ef*-SOD2 activity with a midpoint of ~75 °C. This set of experiments showed that *Ef*-SOD1 enzymes are less stable to temperature than *Ef*-SOD2. We showed previously (Fig. 3) that SOD1s specific activity is affected by metals. Nevertheless, we did not observe any difference in the temperature stability of proteins produced with and without metals in the medium (data not shown). Accordingly, the stability profiles are fully superimposable and in Fig. 4A we show results obtained with proteins produced in the presence of metals only. These data suggest that metals are relevant for specific activity but not for protein stability. One can hypothesize that Cu^2+^ intracellular concentration is insufficient to saturate the overexpressed *Ef-*SOD1s, as reported for other SOD1s^34,35^.

**Figure 5:**
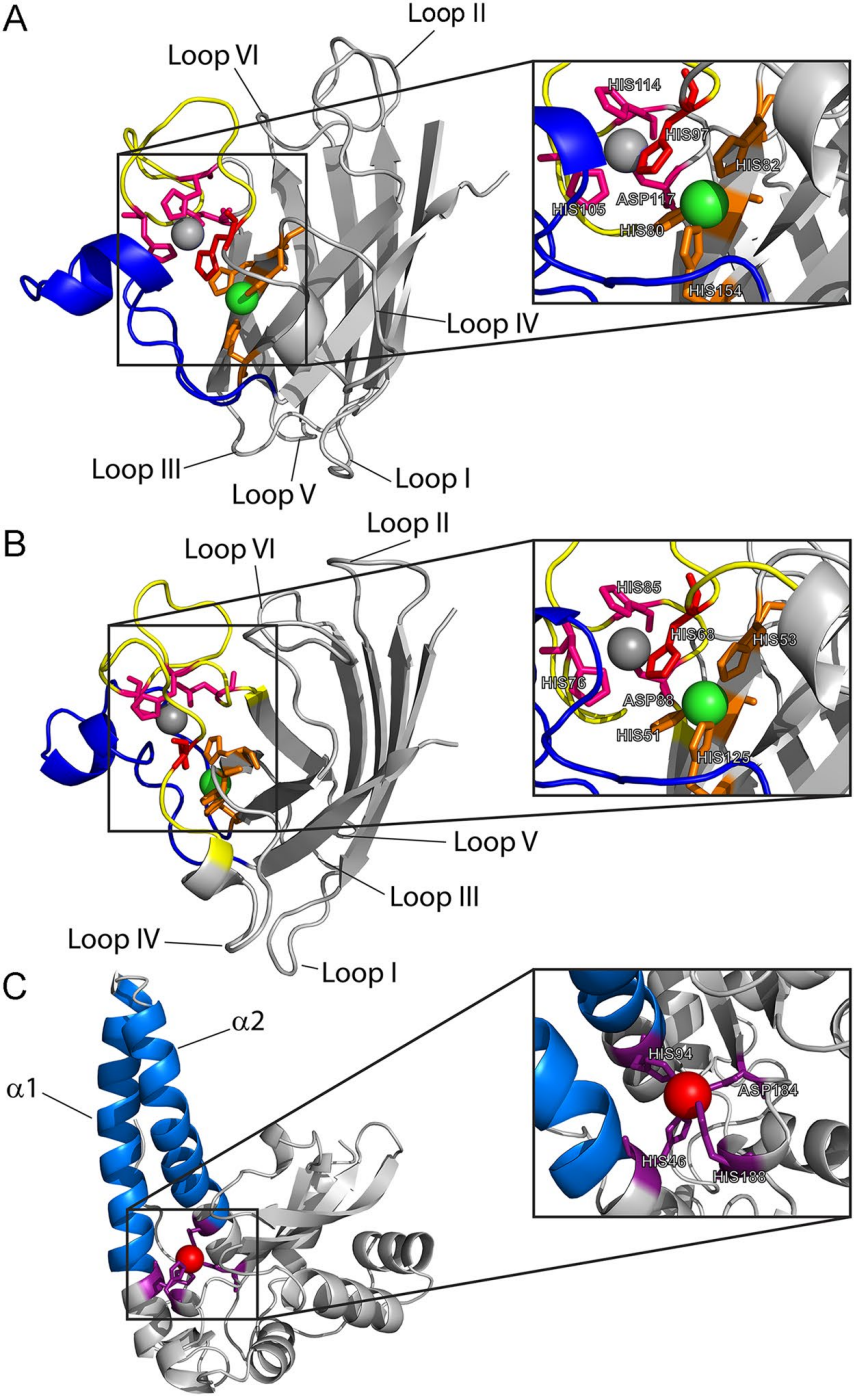
3D models of *Ef*-SODs. *Ef*-SOD1a (**A**) and *Ef*-SOD1b (**B**) were modelled using as the templates *C. elegans* Cu-Zn SOD (PDB:3KBF) and chimeric SOD1 from *Mus musculus* and *Homo sapiens* (PDB: 3LTV). Zinc and copper ions are represented by grey and green spheres, respectively. The Z-loop is highlighted in yellow and the E-loop in blue. Residues involved in the coordination of the zinc ion are in magenta, whereas those that coordinate copper are in orange. The residue of His which coordinate both metal ions is in red. (**C**) 3D structure of *Ef*-SOD2 was modelled using human mitochondrial SOD (PDB:1VAR). The manganese ion is represented by a red sphere and the coordination residues are colored in magenta. Helices α1 and α2 involved in protein oligomerization are highlighted in light blue. 3D structures were modelled with SWISS MODEL^38^.

With this information in our hands, it was of interest to check the impact of temperature on the proteins structure by circular dichroism (CD) analysis. Spectra obtained at 4 °C and 27 °C were identical. As shown in Fig. 4B, the CD spectra of *Ef*-SOD1s were similar with a peak at ~210 nm. This profile is typical of proteins with mainly- β structure and it is reported for other SOD1s^36,37^. The two minima detected at ~208 and ~222 nm reveal high content of α-helix structures in *Ef*-SOD2^Δ^. These data are consistent with the 3D structural model described in a later section (Fig. 5). Thermal stability was investigated by CD analysis at fixed wavelength and temperature raising from 5 °C to 90 °C. The secondary structure of *Ef*-SOD2^Δ^ was unaltered in the range 5 °C– 60 °C. Above 60 °C, the CD signal was rapidly lost with a midpoint temperature (Tm) of 72.8 °C ± 2.1. Above 40 °C a dramatic loss of CD signal was detected in *Ef*-SOD1a^Δ^ and *Ef*-SOD1b samples, with a Tm of 58.4 °C ± 2.3 and 51.3 °C ± 1.5, respectively (Fig. 4C).

### Structural models of *E. focardii* superoxide dismutases

In order to correlate functional analyses with structural information we modeled the 3D structure of *Ef*-SODs with SwissModel^38^ using the following templates: *C. elegans* Cu,Zn SOD1 (PDB: 3KBF) for *Ef*-SOD1a (sequence identity: 45%), chimeric SOD1 from *Mus musculus* and *Homo sapiens* (PDB: 3LTV) for *Ef*-SOD1b (sequence identity: 55%) and human mitochondrial SOD (PDB: 1VAR) for *Ef*-SOD2 (sequence identity: 44%). Templates were chosen based on high sequence identity with the target proteins and the high resolution of available 3D structures. Moreover, the oligomerization state is in agreement with that experimentally determine (see later). The quality of the structural models was estimated using the QMEAN function (Fig. S4).

Structural models of *Ef*-SOD1s show the typical structure of Cu,Zn SODs (Fig. 5A and B) with a β-barrel composed by eight β-sheets containing two Greek key domains and two large loops^13^. Out of these, the zinc-loop (Z-loop) (in yellow in Fig. 5 and in Fig. S3A) is involved in zinc coordination, while the charged residues-rich electrostatic loop (E-loop) (in blue in Fig. 5 and in Fig. S3A) is involved in guiding superoxide to the active site^39^. Loops IV and VI (Figs 5 and S3A) are involved in the oligomerization of human Cu,Zn SOD1^40,41^. Specific residues involved in the coordination of metal ions were identified in the structural models and in sequence alignments (Figs 5 and S3A). Consistent with the model, the Cu^2+^ ion is coordinated by His_80_, His_82_ and His_154_ in *Ef*-SOD1a and by His_51_, His_53_ and His_125_ in *Ef*-SOD1b. Likewise, the Zn^2+^ ion is coordinated by His_105_, His_114_ and Asp_117_ in *Ef*-SOD1a and by His_76_, His_85_ and Asp_88_ in *Ef*-SOD1b. His_97_ and His_68_ bridge Cu^2+^ to a Zn^2+^ ions in *Ef-*SOD1a and *Ef-*SOD1b, respectively. Sequence analysis reveals that the SOD1 E-loop is less conserved in ciliates than in higher eukaryotes, where it usually contains the electrostatic triad and a conserved arginine residue^42^ (Fig. S3A). Among ciliates, the “canonical” electrostatic triad is observed only in *Ot*-SOD1a and *Ot*-SOD1b from *Oxytricha trifallax* and *Sl*-SOD from *Stylonychia lemnae* (Fig. S1), while both *Ef*-SOD1a *and Ef*-SOD1b contain a non-canonical triad (Fig. S3A). In the 3D model of *Ef*-SOD2, two long α-helices at the N-terminus form a helical hairpin structure, while the C-terminus contains a three-stranded β-sheet flanked by four α-helices on both sides (Fig. 5C). The Mn^2+^ ion is coordinated by three His (His_46_, His_94_, His_188_) and one Asp residue (Asp_184_) conserved among MnSODs (Fig. S2 and S3B). In homologous enzymes the helical hairpin structure plays a key role in the formation and stabilization of the tetrameric structure^43,44^. Indeed, in human MnSOD, the substitution of Ile_58_ (boxed in Fig. S3B) with Thr destabilizes the tetramer and promotes a dimeric structure by perturbing the methyl group interaction network and hence impairing the oligomerization interface^44^. We showed by SECS-MALS analysis (Table S2) that *Ef*-SOD2 is tetrameric, although it contains a Thr residue at an equivalent position (Thr_78_, boxed in Fig. S3A). This might indicate that the interaction network involved in oligomerization of the human MnSOD is not conserved in *Ef*-SOD2.

Recombinant *Ef*-SODs, were analyzed by SEC-MALS to determine their molecular weight and oligomerization state (Table S2). The molecular mass of *Ef-*SOD2^Δ^ is close to the theoretical mass of the tetramer, whereas the molecular mass of *Ef*-SOD1b is close to that expected for the dimer. Unfortunately, the high propensity of *Ef*-SOD1a to form aggregates interferes with MALS measurements. The comparison of SEC chromatographic profiles obtained for *Ef*-SOD1a and *Ef*-SOD1b, sharing similar molecular mass, suggests that *Ef*-SOD1a is a dimer because elutes at the same time of *Ef-*SOD1b (data not show).

### Molecular dynamic simulation

**Figure 6:**
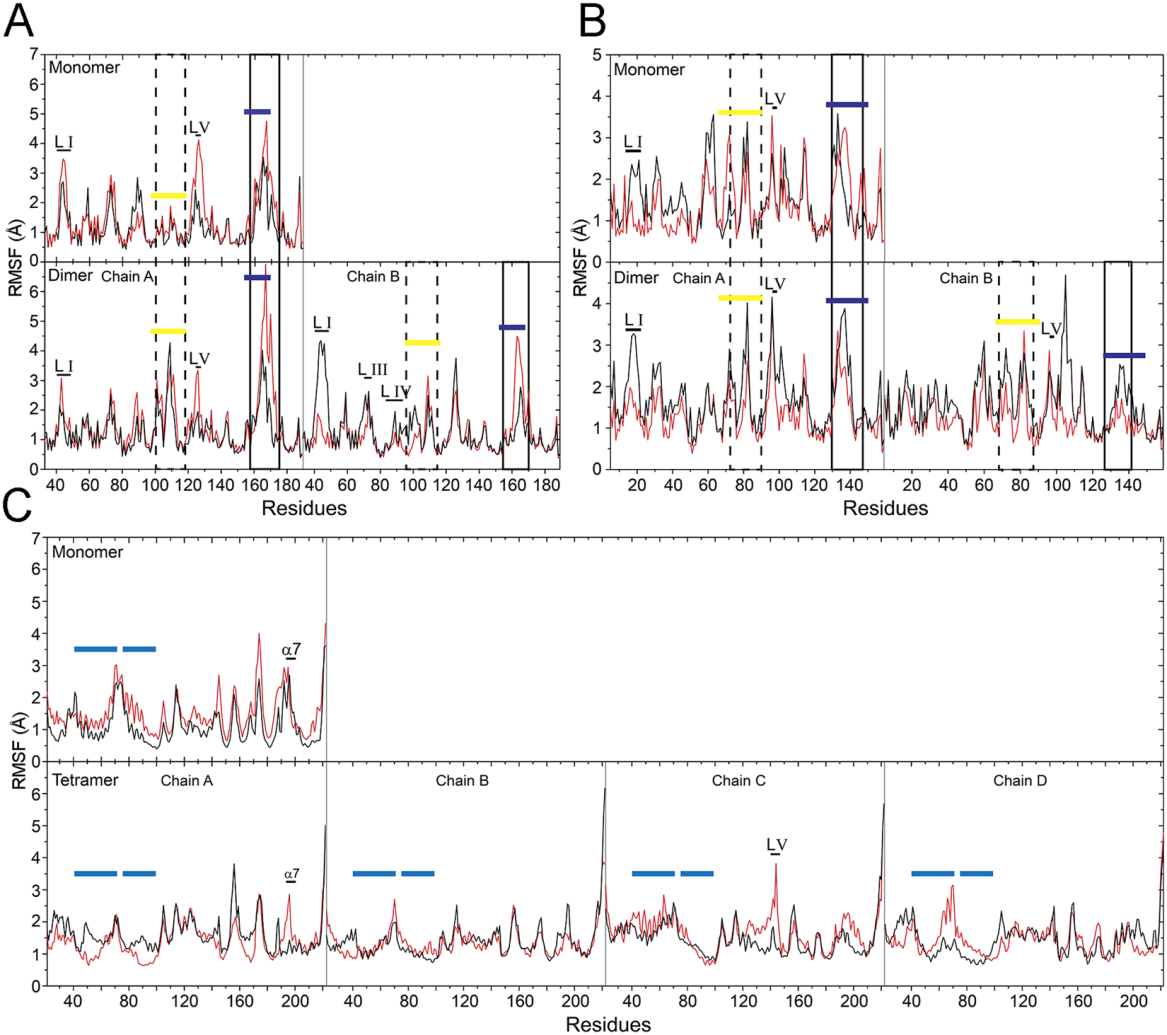
Molecular dynamic simulations of *Ef*-SODs. The flexibility of *Ef*-SODs was evaluated using the RMSF profile. Simulations were carried out at 4 °C (red) and 27 °C (black) for *Ef*-SOD1a (**A**), *Ef-*SOD1b (**B**) and *Ef*-SOD2 (**C**). In *Ef*-SOD1s residues belonging to the Z-Loop and the E-Loop are highlighted in yellow and blue lines, respectively. In *Ef*-SOD2 residues belonging to α1 and α2 are highlighted in light blue lines. Loops with different flexibility at the tested temperatures are indicated.

Most cold active proteins are endowed with higher either local or global flexibility than the mesophilic and thermophilic counterparts to cope with the reduction of dynamics and activity at low temperature^45–47^. Therefore, we set up to investigate *Ef*-SODs by Molecular Dynamic **(**MD) simulations, in which the flexibility of monomeric and oligomeric conformations (dimers for *Ef*-SOD1s and tetramers for *Ef*-SOD2) was compared at 4 °C and 27 °C (Fig. 6), i.e. the same temperatures used for the enzymatic assays.

Flexibility was estimated based on the average root-mean-square fluctuation (RMSF) values recorded for proteins both in the monomeric and the oligomeric form of the enzymes. Due to the limitations inherent to the use of 3D models, in the following we report only relevant differences (>1.5 Å) observed in simulations performed at 4 °C and 27 °C.

In SOD1 enzymes, RMSF differences detected at the two temperatures are small. In particular the E-loop of *Ef*-SOD1a is more flexible at 4 °C than at 27 °C, what might be of importance for catalytic activity in the cold (Fig. 6A). Moreover, loops I and V in the chain A (i.e. the first polypeptide of the dimer) of the dimeric form are highly flexible at 4 °C, whereas loops I of the chain B (i.e. the second polypeptide of the dimer) **is** more flexible at 27 °C.

The RMSF profile of *Ef*-SOD1b shows no relevant differences in flexibility between the monomeric and the dimeric forms at both considered temperatures (Fig. 6B). Indeed, only loop I of chain A and loop V in both chains (Fig. 6B) of the native dimeric conformation are slightly more flexible at 27 °C. However, such loop regions are localised on the opposite sites of the structure with respect to regions involved in subunits interaction^40^, and are not assumed to be involved in protein stability.

In the native tetrameric form of *Ef*-SOD2 but not in the monomer, the α7 helix in chain A, loop V in chain C and residues spanning 66–71 belonging to α1 helix of chain D exhibit higher flexibility at 4 °C (Fig. 6C). The α1 helix is involved in protein oligomerization and may favour the formation of *Ef*-SOD2 tetramers at low temperatures.

Worth of note, in some cases we observed differential effects of temperature among the subunits building the oligomer. These observations may surmise a non-equivalence of the subunits in the quaternary structure or non-symmetric long range effects of oligomerization, suggesting cooperativity of SOD subunits^48^.

To analyse more in depth the effects of temperature on ions-coordinating loops, the flexibility of His and Asp residues involved in ions coordination (i.e. His_80_, His_82_ His_105,_ His_114_, Asp_117_; His_154_ in *Ef*-SOD1a and by His_51_, His_53_ His_76_, Asp_88_ His_85_ and His_125_ in *Ef*-SOD1b and His_46_, His_94_, His_188_, Asp_184_ in *Ef*-SOD2) was evaluated using the root mean square deviation (RMSD) profile (Fig. S5A). Overall, Cu^2+^ coordinating His and Asp residues of *Ef*-SOD1a and *Ef*-SOD1b were slightly more flexible in the dimeric conformation, whereas Mn^2+^ coordinating residues of the *Ef*-SOD2 were more stable in the tetrameric form. The stabilizing effect of oligomerization, reported in several proteins is therefore relevant for the tetrameric SOD2 enzyme but not for the two dimeric enzymes (Fig. S5A).

A key role in the catalytic activity of Cu,Zn SOD is played by Arg residue which follows the electrostatic triad^49^. This residue is conserved in both *Ef*-SOD1s and is found in position 177 and 148 in *Ef*-SOD1a and *Ef*-SOD1b, respectively (Fig. S3A). MD simulations show that temperature and oligomerization state do not change the flexibility of these residues (Fig. S5B), suggesting that catalytic Arg flexibility is nor relevant for the activity of Cu,Zn SODs in the cold.

## Discussion

*E. focardii* is an obligate psychrophilic stenothermal organism restricted to live within a very narrow temperature range. Its optimal growing temperature is 4 °C–5 °C and cell death occurs over 12 °C–15 °C^28^. Besides strict temperature requirements, a major issue for *E*. *focardii* is oxidative stress since it experiences high dissolved oxygen typical of Antarctic marine environments^2^. For these reasons, it allowed us studying the combined effects of oxidative stress and cold on key cell enzymes, superoxide dismutases. Transcriptomic analyses of other Antarctic marine organisms such as notothenioid fishes and the krill *Euphasia superba* show that they overexpress catalases, glutathione peroxidase and SODs under physiological conditions, to avoid cell damages induced by reactive oxygen species (ROS)^50^.

Differently from SOD2, the SOD1 family was believed to be absent in protists^10^ until whole genomes sequencing revealed SOD1 encoding genes in a number of different ciliates^29,51–53^. Recently, SODs from the mesophilic ciliate *Tetrahymena thermophila* were described^29^ showing their importance in the detoxification pathway in continued, elevated presence of metals in the environment. The evolutionary history of SODs is controversial. Fink and Scandalios suggested that bacterial and eukaryotic SOD1 sequences evolved from a common ancestor^54^. Lee and co-authors proposed that SOD1 evolved rapidly in relatively recent times, differently from SOD2, which appear to have evolved at a relatively constant rate over the entire history of eukaryotes^55^. The large differences between eukaryotic and bacterial SOD1s, highlighted also in the phylogenetic tree reported in this paper, do not surmise any obvious common ancestry inside the SOD1 family. Therefore, it was proposed that some eukaryotic Cu,Zn SODs would derive from horizontal gene transfer from the endosymbionts that gave rise to mitochondria^10^. The discovery of the SOD1 family in several ciliates and other protists, including the amoeba *Dictyostelium*, opened new scenarios. The phylogenetic tree reported in this work suggests that bacterial and eukaryotic SOD1s do not derive from a common ancestor. Furthermore, most of the SOD1 isoforms of individual ciliate species do not cluster together but rather belong to separated clades, including the SOD1 isoforms from *Paramecium* and *Tetrahymena*. This result suggests that the evolution of SOD1s may have witnessed several events of gene duplication and diversification, occurred very early in ciliate SOD1 genes, rather than horizontal gene transfer. By contrast, bacterial and eukaryotic SOD2s appear to derive from a common ancestor. Whatever was the evolutionary history, the study of todays *E. focardii* SODs pinpoint some peculiarities in these enzymes that reflect a complex interplay of environmental pressures. Evolution of key cell enzymes is restricted by survival constrains. Nevertheless, gene duplication enlarges the kit of available catalysts and favors diversification. Moreover, SODs under study belong to two different families. Even though we do not have any direct evidence about the sub-cellular localization of the SOD isoenzymes within the ciliate cells, we assume from signal peptide characterized by *in silico* analysis that *Ef*-SOD2 is localized in mitochondria, *Ef*-SOD1a could be either extracellular or membrane bound and *Ef*-SOD1b is cytosolic (given the absence of the sequence of signal peptides).

We report regulation of gene expression by temperature changes permissive for *E. focardii* viability (0 °C and 12 °C). Both *Ef*-SOD1a and *Ef*-SOD1b expression is induced by a mild temperature drop, consistent with similar results from other Antarctic organisms. *Deschampsia antarctica*, a plant adapted to the cold climate, overexpress antioxidant enzymes, including peroxidase, SOD and glutathione reductase that cope with damages by ROS^56,57^. Noteworthy, when this plant was acclimated at 4 °C the expression level of Cu,Zn SODs was higher compared to non-acclimated plants incubated at 13 °C^58^. Similar behaviour was observed for the MnSOD transcription after a mild cold exposure of the Antarctic yeast *Glaciozyma antarctica*^59^. In the bivalve *Yoldia eightsi*, SOD activity is higher at low temperatures^60^. Higher expression of SOD1s during the drop of temperature may be explained by the need to cope with the increased concentration of dissolved oxygen in cool water. Incubation at increased temperature causes a decrease in the mRNA levels of both enzymes that is partly recovered with time. Regulation of *Ef*-SOD2 expression seem to be different. Changes of temperature (both down and up) induce a partial reduction of expression in the short time that is fully recovered within two hours. This would be consistent with the mitochondrial localization of this enzyme suggested by sequence analysis. Mitochondria in fact are exposed to high ROS concentration under any condition.

We studied the temperature dependence of activity and the structural stability of SODs in a broad temperature range (4 °C to 90 °C). It is useful to recall that in most cases the optimal temperature of cold-active enzymes is higher than optimal temperature of organism growth and that an enzyme is classified as “cold active” if it retains activity at low temperature, independently on its optimal temperature^61,62^. All three SODs are active at low temperature. At the same time, they retain high activity upon 20 minutes incubation up to 55/60 °C. This feature is unusual in cold-active enzymes that are often heat sensitive and undergo inactivation and unfolding even at mild temperature. Nevertheless, thermo-tolerance or even thermostability of cold-adapted enzymes was reported previously. For example, Yang and colleagues reported 2.07 hours half-life at 70 °C for a cold-active patatin-like phospholipase from *E.focardii*^33^. Similarly, cold active SODs from Antarctic bacteria such as *Pseudoalteromonas haloplankitis*^63,64^*, Exiguobacterium sp*^65^ and *Aliivibrio salmonicida*^66^ possess high thermal stability associated to catalitic activity in the cold. It has been previously proposed that structural flexibility and rigidity may co-exist in the same molecule in psychrophilic enzymes, because only domains involved in the conformational changes during catalysis need to be flexible^62,67^. This is consistent with the observation that enzyme activity can be impaired by temperature before structural damage occurs^61,62^. However, in our results the Tm describing loss of activity and protein unfolding are very close, and the small differences observed can be accounted for by the different experimental methods employed.

Therefore, we propose that cold activity of *Ef*-SODs is supported by the presence of restricted flexible regions sufficient for low temperature catalysis but not enough extended or mobile to unfold independently of the overall protein structure. Molecular dynamics simulation of the three *Ef*-SODs revealed only minor differences in the temperature-dependent flexibility. This result is not in contrast with our experimental results; on the contrary, it confirms that *Ef*-SODs are indeed flexible at both low and mild temperatures, and that slightly higher local flexibility at 4 °C is sufficient for low temperature catalysis yet maintaining heat tolerance. In conclusion, *Ef*-SODs combine cold activity with thermostability, a characteristic that may be due to the evolutionary origin of this ciliate before Antarctica broke up from Pangaea. In Cenozoic, due to Pangaea fragmentation the complete oceanic circulation around Antarctica and the decreasing atmospheric carbon dioxide concentrations caused a rapid cooling of Antarctica and allowed glaciers to form. Unicellular organisms adapted at higher temperatures, once trapped in the cold Southern Ocean gradually adapted their enzyme structures to the new condition. The combined features of cold activity with overall structural robustness of *Ef*-SODs seem to be functional to confer to key enzymes the ability to be functional under changing environmental conditions and could have been of advantage for the success of this ciliate in the colonization of the Antarctic marine habitat.

## Methods

### Strain and materials

*E. focardii* strains TN1 and TN15 isolated from sediment and seawater samples collected in Antarctica^27^ were cultivated in a cold room at 4 °C in seawater and fed with the green alga *Dunaliella tertiolecta*.

*Escherichia coli* strain DH5 α™ (Invitrogen, Waltham, USA) was the host for plasmid DNA amplification, while strain BL21 (DE3) (EMD Millipore, Billerica, USA) was used for recombinant protein expression. Random hexamer primers for cDNA synthesis were from Thermo Fisher Scientific (Thermo Fisher Scientific, Waltham, MA USA) and oligonucleotides from Metabion (Metabion International AG, Steinkirchen, Germany). Q5® High-Fidelity DNA Polymerase was purchased from New England Biolabs (New England Biolabs, Ipswich, MA). Materials for SOD activity assay (cytochrome C, xanthine and xanthine oxidase), TRIzol reagent were from Sigma-Aldrich (Saint Louis, Missouri, USA). 2X SYBR Green Mix was purchased from Carlo Erba (Milan, Italy).

### RNA Extraction and cDNA synthesis

Cell cultures of *E. focardii* constantly grown at 4 °C were incubated at 0 °C, 4 °C and 12 °C, for 1 and 2 hours. Total RNA was extracted from ~20000 cells (representing a mix of both TN1 and TN15 strains) using TRIzol reagent (Sigma, Milan, Italy) according to manufacturer’s instructions. Isolated RNA was resuspended in diethylpyrocarbonate treated water (Sigma) and subjected to DNase I (from Carlo Erba, Milan Italy) treatment to remove genomic DNA. Total RNA concentration was measured at 260 nm with UV spectrophotometer (UV 1600PC). RNA integrity was verified by denaturing electrophoresis of a 2 µg sample on 1% agarose gel. DNase I-treated RNA samples were used as the template to amplify the *E. focardii* SSUrRNA gene to verify the absence of genomic DNA contamination. First strand cDNA was synthesized at 42 °C for 1 h from 2 µg of total RNA using random hexamer primers (5 ng/µl final concentration) and Moloney murine leukemia virus reverse transcriptase (Lucigen, Middleton, WI, USA).

### Quantitative real time PCR

The relative expression patterns of *Ef*-SODs coding genes were measured in cDNA samples from control (4 °C) and stressed (0 °C and 12 °C) cells by comparative-threshold qPCR using the SYBR green DNA-binding method^68^. Quantitative real time PCR analysis was carried out in a final volume of 25 µl containing 12.5 µl of 2X SYBR Green Mix (Carlo Erba, Milan Italy), 10 µM of forward and reverse primers (Table S3), 1 µl of cDNA template (100 ng/µl) and 9.5 µl of nuclease-free water. The *E. focardii* SSUrDNA (GenBank ID**:** EF094961) gene was used for normalization. Amplification reactions were performed in triplicate in a Multicolor qPCR MX3000P thermocycler (Stratagene, Milan, Italy) as follows: 95 °C for 2 min; 40 cycles (95 °C for 15 s, 55 °C for 1 min) and a final cycle (95 °C for 1 min, 55 °C for 30 s, 95 °C for 30 s).

The relative ratio of each *Ef*-SOD gene was determined according to^69^, by estimating the ∆∆CT, it correspond to

$${\rm{\Delta }}{\rm{\Delta }}\mathrm{CT}={{\rm{\Delta }}\mathrm{CT}}_{{\rm{unknown}}}-{{\rm{\Delta }}\mathrm{CT}}_{{\rm{control}}}$$

where ∆CT is the difference between the mean Ct of the target gene and the mean Ct of housekeeping gene. Ct is the PCR cycle number at which fluorescent signal is above the threshold that is set to exclude the fluorescent signal background. ∆Ct is the Ct deviation of the control minus the sample of the target (SOD) or reference (housekeeping) gene. The unknown and control ∆Cts in the ∆∆CT formula stand for stress and physiological condition respectively.

### Sequence analysis

The amino acid sequences of *Ef*-SODs were deduced by the Expert Protein Analysis System (http://www.expasy.org/). The theoretical molecular weight was determined by Expasy-ProtParam online tool (http://www.expasy.org/tools/protparam.html). Signal peptides were predicted using PROTTER ver.1.0^30^ and MitoFates^31^. Multiple alignments were performed by Clustal Omega. The phylogenetic tree was constructed by the Neighbour Joining algorithm^70^ using MEGA5^71^.

### Cloning of *E. focardii* superoxide dismutases sequences

Synthetic genes optimized for expression in *E. coli* (Genscript, Piscataway, NJ, USA) were cloned in frame with a C-terminal 6xHis-Tag into pET-21a vector (EMD, Millipore, Billerica, MA, USA) between *NdeI* and *XhoI* sites to obtain, pET-21a [*Ef*-SOD1a], pET-21a [*Ef*-SOD1b] and pET-21a [*Ef*-SOD2].

QuickChange® PCR was performed to remove signal peptide sequences from *Ef*-SOD2 and *Ef*-SOD1a. Forward and reverse primer sequences are reported in Table S3. Reactions were carried out using Q5® High-Fidelity DNA Polymerase and Eppendorf Master-cycler (Eppendorf, Netheler Hinz Gmbh, Hamburg, Germany) under the following conditions: 1 cycle (98 °C for 2 min), 25 cycles (98 °C 10 sec, 59 °C 25 sec for *Ef*- SOD2 or 51 °C 25 sec for *Ef*-SOD1a and 72 °C 180 sec), and a final cycle at 72 °C for 3 min. The deletion of signal peptide in pET-21a [*Ef*-SOD2^Δ^] and in pET-21a [*Ef*-SOD1a^Δ^] was assessed by enzyme restriction and by DNA sequencing.

### Recombinant proteins production and purification

Recombinant proteins produced by cells grown in Zym-5052 medium^72^ were extracted as described^73^. To evaluate the effect of metal cofactors on activity, copper (CuSO_4_) and zinc (ZnCl_2_) were added at 250 µM final concentration to cultures producing *Ef*-SOD1s, whereas manganese (MnSO_4_) were added at the final concentration of 250 µM to *Ef*-SOD2 producing cultures.

Proteins were purified at 4 °C by metal ion affinity chromatography on a nickel-nitrilotriacetic acid agarose resin (Jena bioscience, Germany). Samples containing protein at the highest concentration were pooled and buffer exchanged twice by gel filtration on PD10 columns (GE healthcare) against 200 mM sodium phosphate buffer, pH 7.0.

Protein concentration was determined by the Bradford protein assay (Bio-Rad, California, USA), using bovine serum albumin as a standard.

### Activity assays

Enzyme activity was assayed according to McCord and Fridovich^8^ using a Jasco V-530 UV/VIS spectrophotometer (JASCO International Co. Ltd., Hachioji, Tokyo, Japan). The activity assay was performed at 4 and 27 °C with *Ef*-SODs produced either in the absence or in the presence of the respective metal cofactor in the culture medium. Experiments were in triplicate. One unit of SOD was defined as the amount of protein required to inhibit the reduction of cytochrome c by the superoxide radical by approximately 50%^8^.

To study the effect of temperature on *Ef*-SODs activity, proteins were incubated 20 minutes at temperatures in the range 5 °C–90 °C and then assayed for activity at room temperature. Experiments were in triplicate.

### CD spectroscopy

CD spectra of 2 µM *Ef*-SODs were measured with a J-815 spectropolarimeter (Jasco Corp., Easton, MD, USA), using 0.1 cm path-length cuvette. Measurements were performed in the range 190–260 nm, with 0.2-nm data pitch and 20-nm/min scanning speed. All spectra were corrected for buffer contribution, averaged from two independent acquisitions, and smoothed by using a third-order least square polynomial fit.

Thermal denaturation spectra were obtained measuring the CD signal in correspondence of the following minimum peaks: 205 nm for *Ef*-SOD1a^Δ^, 215 nm for *Ef*-SOD1b, 210 nm and 208 nm for *Ef*-SOD2^Δ^ fixed wavelength when progressively heating the sample from 5 °C to 90 °C. Measurements were performed in triplicate with a data pitch of 2 °C and a temperature slope of 0.5 °C/min.

### SEC-MALS

Oligomerization was studied by SEC-MALS analysis. 200 µl of protein (1 mg/ml) was injected onto Superose12 10/300 GL (GE Healthcare) and eluted with a mobile phase of 50 mM sodium phosphate buffer, NaCl 150 mM, pH 7.5. Light scattering and refractive index were measured with a Wyatt Dawn Heleos detector (Wyatt Technology Corporation, Santa Barbara, USA).

### Protein modelling and simulations

3D structures were modeled through the SwissModel server^38^. *Ef*-SOD1a was modeled based on the structure of *C. elegans* Cu,Zn SOD1 (PDB: 3KBF, 45% sequence identity, resolution: 1.3 Å and 81% of coverage with *Ef*-SOD1a), *Ef*-SOD1b based on the structure of the chimeric SOD1 from *Mus musculus* and *Homo sapiens* (PDB: 3LTV; 55% sequence identity, resolution: 2.45 Å and 93% of coverage with *Ef*-SOD1b)^74^, *Ef*-SOD2 was modeled based on the structure of human mitochondrial SOD (PDB:1VAR; 44% identity, resolution: 2.45 Å and 89% of coverage with *Ef*-SOD2)^43^. Models were obtained in the dimeric form for *Ef*-SOD1s, and in the tetrameric form for *Ef*-SOD2.

In order to compare the effect of temperature and oligomerization status on structure flexibility, molecular dynamic (MD) simulations were performed. 3D Models complexed with Cu, Zn (*Ef*-SOD1s) or Mn (*Ef*-SOD2) were subjected to 100 ns MD simulations at either 227.15 K (4 °C) or 300.15 K (27 °C) under constant temperature and pressure (1 atm). Analysis was performed on both monomers and dimers (*Ef*-SOD1s) or tetramers (*Ef*-SOD2). MD simulations were performed with the GPU implementation of the pmemd code^75^ from AMBER14^76^ employing the ff99SSBildn forcefield^77^.

Root mean square fluctuation (RMSF), the average residues flexibility during the trajectory and the root mean square deviation (RMSD) of particular residues, like ions binders, were evaluated in order to analyze temperature and oligomerization status effect on protein structure.

## Electronic supplementary material

Supplementary Materials

## References

[1] Marx JC, Collins T, D’Amico S, Feller G, Gerday C (2007). Cold-adapted enzymes from marine Antarctic microorganisms. Marine Biotechnology.

[2] Lesser MP (2006). Oxidative stress in marine environments: biochemistry and physiological ecology. Annu. Rev. Physiol..

[3] Hochachka PW, Somero GN (1968). The adaptation of enzymes to temperature. Comparative biochemistry and physiology.

[4] D’Amico S, Collins T, Marx JC, Feller G, Gerday C (2006). Psychrophilic microorganisms: challenges for life. Embo Reports.

[5] De Maayer, P., Anderson, D., Cary, C. & Cowan, D. A. Some like it cold: understanding the survival strategies of psychrophiles. *EMBO reports*, e201338170 (2014). 10.1002/embr.201338170 PMC4210084 24671034

[6] Fang FC (2004). Antimicrobial reactive oxygen and nitrogen species: concepts and controversies. Nature Reviews Microbiology.

[7] Margesin R, Miteva V (2011). Diversity and ecology of psychrophilic microorganisms. Research in microbiology.

[8] McCord JM, Fridovich I (1969). Superoxide dismutase an enzymic function for erythrocuprein (hemocuprein). Journal of Biological chemistry.

[9] McCord JM, Fridovich I (1988). Superoxide dismutase: the first twenty years (1968–1988). Free Radical Biology and Medicine.

[10] Miller A-F (2012). Superoxide dismutases: ancient enzymes and new insights. FEBS letters.

[11] Benov LT, Fridovich I (1994). *Escherichia coli* expresses a copper-and zinc-containing superoxide dismutase. Journal of Biological Chemistry.

[12] Steinman HM, Ely B (1990). Copper-zinc superoxide dismutase of Caulobacter crescentus: cloning, sequencing, and mapping of the gene and periplasmic location of the enzyme. Journal of bacteriology.

[13] Tainer JA, Getzoff ED, Beem KM, Richardson JS, Richardson DC (1982). Determination and analysis of the 2 Å structure of copper, zinc superoxide dismutase. Journal of molecular biology.

[14] Antonyuk SV, Strange RW, Marklund SL, Hasnain SS (2009). The structure of human extracellular copper–zinc superoxide dismutase at 1.7 Å resolution: insights into heparin and collagen binding. Journal of molecular biology.

[15] Marklund SL (1984). Extracellular superoxide dismutase and other superoxide dismutase isoenzymes in tissues from nine mammalian species. Biochemical Journal.

[16] Bannister JV, Bannister WH, Rotilio G (1987). Aspects of the structure, function, and applications of superoxide dismutas. Critical Reviews in Biochemistry.

[17] James ER (1994). Superoxide dismutase. Parasitology Today.

[18] Fréalle E (2006). Manganese superoxide dismutase based phylogeny of pathogenic fungi. Molecular phylogenetics and evolution.

[19] Sugio S, Hiraoka BY, Yamakura F (2000). Crystal structure of cambialistic superoxide dismutase from Porphyromonas gingivalis. The FEBS Journal.

[20] Huang J-K, Wen L, Ma H, Huang Z-X, Lin C-T (2005). Biochemical characterization of a cambialistic superoxide dismutase isozyme from diatom Thallassiosira weissflogii: cloning, expression, and enzyme stability. Journal of agricultural and food chemistry.

[21] Santos R, Bocquet S, Puppo A, Touati D (1999). Characterization of an atypical superoxide dismutase from Sinorhizobium meliloti. Journal of bacteriology.

[22] Jackson SMJ, Cooper JB (1998). An analysis of structural similarity in the iron and manganese superoxide dismutases based on known structures and sequences. Biometals.

[23] Lah MS (1995). Structure-function in *Escherichia coli* iron superoxide dismutase: comparisons with the manganese enzyme from Thermus thermophilus. Biochemistry.

[24] Parker MW, Blake CCF (1988). Crystal structure of manganese superoxide dismutase from Bacillus stearothermophilus at 2.4 Å resolution. Journal of molecular biology.

[25] Smith MW, Doolittle RF (1992). A comparison of evolutionary rates of the two major kinds of superoxide dismutase. Journal of molecular evolution.

[26] Hong-Duk Y, Eun-Ja K, Jung-Hye R, Hah YC, Sa-Ouk K (1996). A novel nickel-containing superoxide dismutase from Streptomyces spp. Biochemical Journal.

[27] Valbonesi A, Luporini P (1993). Biology of Euplotes focardii, an Antarctic ciliate. Polar Biology.

[28] Pucciarelli S (2009). Molecular cold-adaptation of protein function and gene regulation: The case for comparative genomic analyses in marine ciliated protozoa. Marine Genomics.

[29] Ferro D (2015). Cu, Zn superoxide dismutases from Tetrahymena thermophila: molecular evolution and gene expression of the first line of antioxidant defenses. Protist.

[30] Omasits U, Ahrens CH, Müller S, Wollscheid B (2013). Protter: interactive protein feature visualization and integration with experimental proteomic data. Bioinformatics.

[31] Fukasawa Y (2015). MitoFates: improved prediction of mitochondrial targeting sequences and their cleavage sites. Molecular & Cellular Proteomics.

[32] Yang G (2013). Characterization and comparative analysis of psychrophilic and mesophilic alpha-amylases from Euplotes species: a contribution to the understanding of enzyme thermal adaptation. Biochemical and biophysical research communications.

[33] Yang G (2013). Characterization of the first eukaryotic cold-adapted patatin-like phospholipase from the psychrophilic Euplotes focardii: identification of putative determinants of thermal-adaptation by comparison with the homologous protein from the mesophilic Euplotes crassus. Biochimie.

[34] Eiamphungporn W, Yainoy S, Prachayasittikul V (2016). Enhancement of Solubility and Specific Activity of a Cu/Zn Superoxide Dismutase by Co-expression with a Copper Chaperone in *Escherichia coli*. Iranian Journal of Biotechnology.

[35] Ahl M, Lindberg MJ, Tibell LAE (2004). Coexpression of yeast copper chaperone (yCCS) and CuZn-superoxide dismutases in *Escherichia coli* yields protein with high copper contents. Protein expression and purification.

[36] Potter SZ (2007). Binding of a single zinc ion to one subunit of copper− zinc superoxide dismutase apoprotein substantially influences the structure and stability of the entire homodimeric protein. Journal of the American Chemical Society.

[37] Stevens JC (2010). Modification of superoxide dismutase 1 (SOD1) properties by a GFP tag–implications for research into amyotrophic lateral sclerosis (ALS). PLoS One.

[38] Biasini M (2014). SWISS-MODEL: modelling protein tertiary and quaternary structure using evolutionary information. Nucleic Acids Research.

[39] Fetherolf MM, Boyd SD, Winkler DD, Winge DR (2017). Oxygen-dependent activation of Cu, Zn-superoxide dismutase-1. Metallomics.

[40] Banci L (1998). Solution structure of reduced monomeric Q133M2 copper, zinc superoxide dismutase (SOD). Why is SOD a dimeric enzyme?. Biochemistry.

[41] Banci L, Bertini I, Cramaro F, Del Conte R, Viezzoli MS (2002). The solution structure of reduced dimeric copper zinc superoxide dismutase. The FEBS Journal.

[42] Fisher CL (1997). Computational, pulse‐radiolytic, and structural investigations of lysine‐136 and its role in the electrostatic triad of human C u, Z n superoxide dismutase. Proteins: Structure, Function, and Bioinformatics.

[43] Borgstahl GEO (1992). The structure of human mitochondrial manganese superoxide dismutase reveals a novel tetrameric interface of two 4-helix bundles. Cell.

[44] Borgstahl GEO (1996). Human mitochondrial manganese superoxide dismutase polymorphic variant Ile58Thr reduces activity by destabilizing the tetrameric interface. Biochemistry.

[45] Georlette D (2004). Some like it cold: biocatalysis at low temperatures. FEMS microbiology reviews.

[46] Pucci F, Rooman M (2017). Physical and molecular bases of protein thermal stability and cold adaptation. Current opinion in structural biology.

[47] Åqvist J, Isaksen GV, Brandsdal BO (2017). Computation of enzyme cold adaptation. Nature Reviews Chemistry.

[48] Sheng Y (2014). Superoxide dismutases and superoxide reductases. Chemical reviews.

[49] Falconi M, Melchionna S, Desideri A (1999). Molecular dynamics simulations of Cu, Zn superoxide dismutase: effect of temperature on dimer asymmetry. Biophysical chemistry.

[50] Clark MS (2011). Antarctic krill 454 pyrosequencing reveals chaperone and stress transcriptome. PLos one.

[51] Lobanov AV (2017). Position-dependent termination and widespread obligatory frameshifting in Euplotes translation. Nature structural & molecular biology.

[52] Swart EC (2013). The Oxytricha trifallax macronuclear genome: a complex eukaryotic genome with 16,000 tiny chromosomes. PLoS biology.

[53] Arnaiz O, Sperling L (2010). ParameciumDB in 2011: new tools and new data for functional and comparative genomics of the model ciliate Paramecium tetraurelia. Nucleic acids research.

[54] Fink RC, Scandalios JG (2002). Molecular evolution and structure–function relationships of the superoxide dismutase gene families in angiosperms and their relationship to other eukaryotic and prokaryotic superoxide dismutases. Archives of Biochemistry and Biophysics.

[55] Lee YM, Friedman DJ, Ayala FJ (1985). Superoxide dismutase: an evolutionary puzzle. Proceedings of the National Academy of Sciences.

[56] Echauri SAG (2009). Heterologous expression of a novel psychrophilic Cu/Zn superoxide dismutase from Deschampsia antarctica. Process Biochemistry.

[57] Pérez-Torres E (2004). The role of photochemical quenching and antioxidants in photoprotection of Deschampsia antarctica. Functional Plant Biology.

[58] Sánchez-Venegas JR, Dinamarca J, Moraga AG, Gidekel M (2009). Molecular characterization of a cDNA encoding Cu/Zn superoxide dismutase from Deschampsia antarctica and its expression regulated by cold and UV stresses. BMC research notes.

[59] Boo SY (2013). Thermal stress responses in Antarctic yeast, Glaciozyma antarctica PI12, characterized by real-time quantitative PCR. Polar biology.

[60] Abele D, Tesch C, Wencke P, Pörtner HO (2001). How does oxidative stress relate to thermal tolerance in the Antarctic bivalve Yoldia eightsi?. Antarctic Science.

[61] Feller G, Gerday C (2003). Psychrophilic enzymes: hot topics in cold adaptation. Nature reviews microbiology.

[62] Feller G (2010). Protein stability and enzyme activity at extreme biological temperatures. Journal of Physics: Condensed Matter.

[63] Castellano I (2006). Psychrophilic superoxide dismutase from Pseudoalteromonas haloplanktis: biochemical characterization and identification of a highly reactive cysteine residue. Biochimie.

[64] Merlino A (2010). Structure and flexibility in cold-adapted iron superoxide dismutases: the case of the enzyme isolated from Pseudoalteromonas haloplanktis. Journal of structural biology.

[65] Nonaka K, Yoon K-S, Ogo S (2014). Biochemical characterization of psychrophilic Mn-superoxide dismutase from newly isolated Exiguobacterium sp. OS-77. Extremophiles.

[66] Pedersen HL, Willassen NP, Leiros I (2009). The first structure of a cold-adapted superoxide dismutase (SOD): biochemical and structural characterization of iron SOD from Aliivibrio salmonicida. Acta Crystallographica Section F: Structural Biology and Crystallization Communications.

[67] Lonhienne T, Gerday C, Feller G (2000). Psychrophilic enzymes: revisiting the thermodynamic parameters of activation may explain local flexibility. Biochimica et Biophysica Acta (BBA)-Protein Structure and Molecular Enzymology.

[68] Morrison TB, Weis JJ, Wittwer CT (1998). Quantification of low-copy transcripts by continuous SYBR Green I monitoring during amplification. Biotechniques.

[69] Pfaffl MW (2001). A new mathematical model for relative quantification in real-time RT–PCR. Nucleic acids research.

[70] Saitou N, Nei M (1987). The neighbor-joining method: a new method for reconstructing phylogenetic trees. Molecular biology and evolution.

[71] Tamura K (2011). MEGA5: molecular evolutionary genetics analysis using maximum likelihood, evolutionary distance, and maximum parsimony methods. Molecular biology and evolution.

[72] Studier FW (2005). Protein production by auto-induction in high-density shaking cultures. Protein Expression and Purification.

[73] Brocca S (2016). A bacterial acyl aminoacyl peptidase couples flexibility and stability as a result of cold adaptation. The FEBS journal.

[74] Seetharaman SV, Taylor AB, Holloway S, Hart PJ (2010). Structures of mouse SOD1 and human/mouse SOD1 chimeras. Archives of biochemistry and biophysics.

[75] Salomon-Ferrer R, Götz AW, Poole D, Le Grand S, Walker RC (2013). Routine microsecond molecular dynamics simulations with AMBER on GPUs. 2. Explicit solvent particle mesh Ewald. Journal of chemical theory and computation.

[76] Case, D. A. *et al*. Amber 14 (2014).

[77] Lindorff‐Larsen K (2010). Improved side‐chain torsion potentials for the Amber ff99SB protein force field. Proteins: Structure, Function, and Bioinformatics.

