# Supplementary material for: Antarctic marine ciliates under stress: superoxide dismutases from the psychrophilic Euplotes focardii are cold-active yet heat tolerant enzymes
Source: Sci Rep. 2018 Oct 3;8:14721. doi: 10.1038/s41598-018-33127-1 (PMC6170424; doi:10.1038/s41598-018-33127-1)
Supplement: Supplementary file 1 — Supplementary Materials [file 41598_2018_33127_MOESM1_ESM.pdf]

Supplementary materials

Title page:

Antarctic marine ciliates under stress: superoxide dismutases from the psychrophilic *Euplotes focardii* are cold-active yet heat tolerant enzymes

Alessandro Pischedda[1], Kesava Priyan[2], Marco Mangiagalli[1], Federica Chiappori[3], Luciano Milanesi[3], Cristina Miceli[2], Sandra Pucciarelli[2*], Marina Lotti[1]

[1]*Department of Biotechnology and Biosciences, University of Milano-Bicocca, Piazza della Scienza 2, 20126 Milano, Italy*

[2]*School of Biosciences and Veterinary Medicine, University of Camerino, Via Gentile III da Varano, 1, 62032, Camerino (MC), Italy*

[3]*Institute of Biomedical Technologies – CNR, Segrate (Mi), Italy*

* Corresponding Author

```
Ef-SOD1a        MLAYFVLPSSAALLSY-----TMSKNRIGDCEKGDTEKKAICLVNPEKNQVAKGIVHFEQ    55
Sl-SOD1         MASKFLLFTMGALPLAQLQR-QQFSQNNEMAEEGNTERQAICILNSENGQTGKGIVHFIQ    59
Ot-SOD1a        MASRFLMLSLGAYAYNRQQTSNQANQHSEMEATEDAPRQAICILNAEKGQTAHGVVHFLQ    60
Ot-SOD1b        MASRFLMLSLGAYAYNRQQTSNQANQHSEMEATEDAPRQAICILNAEKGQTAHGVVHFLQ    60
Ot-SOD1c        -----------------------------MEGKTTVTHYAVCLMQEDHHSGVSGTVKFMQ    31
Ot-SOD1d        ------------------------------------------------------------    0
Tt-SOD1a        --------AA------GTALFSYSNQKTYKAEKNAGERIAIAILYPAPGYDVTGAVTFYQ    46
Pt-SOD1a        ------------------------------------------------------------    0
Ef-SOD1b        --------------------------------MEATAAYALCILRPDGGSSVNGVVRFIQ    28
Pt-SOD1b        ------------------------------------------------------------    0
Tt-SOD1c        ------------------------------------------------------------    0
Im-SOD1a        -------------------------------MADTAPIYAICILNPDGGSGVSGLVKLVQ    29
Im-SOD1b        -------------------------------MQNNLPLYATCILSPDGNSGVNGLVKLIQ    29

Ef-SOD1a        ENQYAKTHIFGNFTNLSKNHAHGFHIHVYGNLSKGCLTAGPHYNPYAKEHGGPHSTVRHV    115
Sl-SOD1         SSLVARTQIQGQFSGLNPNQRHGFHIHQYGNLSQGCVTAGPHYNPLNQVHGGPDSAIRHV    119
Ot-SOD1a        NSMFQKTRIQGEFQGLTPSHKHGFHIHQYGNLSQGCVTAGPHFNPLNQLHGGPDSIIRHV    120
Ot-SOD1b        NSMFQKTRIQGEFQGLTPSHKHGFHIHQYGNLSQGCVTAGPHFNPLNQLHGGPDSIIRHV    120
Ot-SOD1c        DEGG-RVRISAQLT-GLKPGLHGFHVHQFGNLTNGCVTAGAHFNPHKKTHAGPKDENRHV    89
Ot-SOD1d        -----------------------------------------HYNPHKKTHAGPKDENRHV    19
Tt-SOD1a        KDLHSKTQITARLKNLNPNGLFGFHIHEFGDLTNGTESVGPHYNPFNKKHGSPREDESHM    106
Pt-SOD1a        ------------------NSLHGVHIHEFGDLSNGCATAGPHFNPFEQEHGGPLDEKRHV    42
Ef-SOD1b        QAGG-KTRVIAEIT-GLPAGLHGFHVHKFGNLIEGCKTAGPHFNPHGKEHGGPLSEERHV    86
Tt-SOD1b        --------------------------HEYGNLIDGCKSAGAHFNPTKQTHGAPDSKERHV    34
Pt-SOD1b        ----------------LSDGLHGFHIHEFGNLIKGCITAGPHYNPHGKLHGGPKDQERHV    44
Tt-SOD1c        -----------------------------------------HFNPFNKQHGGPNDENRHV    19
Im-SOD1a        QG-D-QVTITATVN-GLKTGLHGFHIHQFGNLTEGCKTAGPHFNPFQKTHGGPHDVERHV    86
Im-SOD1b        QG-D-YTTITATIN-GLKKGLHGFHIHQFGNLTEGCKTAGPHFNPFNSTHGGPQDTERHV    86
                                                         *:**  . *..* .   *:

Ef-SOD1a        GDLGNVFSD--SEGNASFDHWDPQIALSGS-NSVIGRACVLHKFTDDHGCGGNGESKKTG    172
Sl-SOD1         GDLGNVQAD--ESGNAKVDFIDHQVTLYGP-HSVVGRACVLHRDTDDLGTSDNEESKKTG    176
Ot-SOD1a        GDL---QAD--DQGLSKLDFEDHQITLHGP-LSIVGRACVLHRDTDDHGTADNEESKKTG    177
Ot-SOD1b        GDLGNVQSD--EQGLSKVDFEDHQITLHGP-LSIVGRACVLHRDTDDYGTADNEESKKTG    177
Ot-SOD1c        GDLGNIEVG--ADGVGKFDMDDDLIMIYGADNNIIGRAMVVHAQEDDLGRGGNEESLITG    147
Ot-SOD1d        GDLGNIEVG--ADGVGKFDMDDDLIMIYGADNNIIGRAMVVHAQEDDLGRGGNEESLITG    77
Tt-SOD1a        GDLGNIKAD--DLGYGYYTSENNKVTLFGE-YSVVGRSVLVNKNEDDLGRGNHPDSHTNG    163
Pt-SOD1a        GDLGNIKTD--ERGNGYLAYEDNQIQLYGE-YSILGRSVVVHAGQDDLGRGNQKDSKTTG    99
Ef-SOD1b        GDMGNVDAG--EDGVAKLDYEDAQIELIGE-HSIIGRSVVCHAGTDDHGEGGHDDSKKTG    143
Tt-SOD1b        GDLGNIENKLSEENVAVYEIVDHLISLYGE-YNVIGRSCVIHADEDDLGLGNFEDSKTTG    93
Pt-SOD1b        GDLGNVHSE---NGVAHFKINDDFVKLSGE-FSVIGRSMVVHANEDDLGKSDHPDSKSTG    100
Tt-SOD1c        GDLGNVTAV-D-GQDTNFEFQSDLIRLSGE-NTIVGRSFVIHADEDDLGKGNFEDSKTTG    76
Im-SOD1a        GDLGNIQAV-E-GQQAQFSIVDKLIKLDGA-NSVLGRSFVVHADEDDLGKGGHDDSKTTG    143
Im-SOD1b        GDLGNVETL-EDGQTTHFKIVDKLIKLDGL-NSVLGRSFVIHADQDDLGKGNFEDSKTTG    144
                **:**:               .  : : *   .::**: :   ** * ..  :*  .*

Ef-SOD1a        NAGPRIGCGVIGLDA---------    187
Sl-SOD1         NAGPRIACGIIGLSKE--------    192
Ot-SOD1a        NAGPRIACGIIGLSKE--------    193
Ot-SOD1b        NAGPRIACGIIGLSKE--------    193
Ot-SOD1c        NAGGRLACGVIGLSGPISM-----    166
Ot-SOD1d        NAGGRLACGVIGLSGPISM-----    96
Tt-SOD1a        HSGPRIAAGIIGLAYELKNLPARF    187
Pt-SOD1a        NSGARLACGVIGLASGFKNLQPYK    123
Ef-SOD1b        HAGARLACGTIGLSDTFDV-----    162
Tt-SOD1b        HAGARVACGPIGLCAKFSFDF---    114
Pt-SOD1b        NAGARLACGVIGISGPFEFD----    120
Tt-SOD1c        HAGARLACGIIALAAPFENF----    96
Im-SOD1a        HAGARLACGTIGLSGPF-------    160
Im-SOD1b        HAGARLACCTIGLSGPF-------    161
                ::* *:..  *.:
```

Figure S1. Sequence alignment of SOD1s from ciliate species. Alignment was obtained by ClustalOmega. Residues involved in the coordination of Cu and Zn are highlighted with the same color code used in Figure 5. Residues of the electrostatic triad are in blue, Arg residue following the electrostatic triad is in brown.

```
Human MnSOD     MLSRAVCGTSR--QLAPALGYLGSRQKHSLPDLPYDYGALEPHINAQIMQLHHSKHHAAY      58
Ef-SOD2         MLNRVIYKRSQ-ML---FSRAF--SSKVELPALPWEISSLEPTLSAYLLDFHYNKHHQTY      54
Tt-SOD2         ------------------------------------------------------------      0
Ot-SOD2         MLNKAIQNCRQNGLFIQTARCFSSTKKAELKPLPWDINALEPVLSGNLLDHHYNRHHKLY      60
Sl-SOD2        ------------------------------------------------------------ 0

Human MnSOD     VNNLNVTEEKYQEALAKGDVTAQTALQPALKFNGGGHINHSIFWTNLSPNG--GGE---P      113
Ef-SOD2         VNNLNSLLQQEGEAIEKGDFETATNLAPLIRFHGGGHINHTFFWHTLASKSQGGGERPSD      114
Tt-SOD2         --------------------HKIAQLQSGLRFNLGGHINHAIYWDNLAPVSRGGGVFPDQ      40
Ot-SOD2         VTKFNETLDQLDEAAAKGDHAKIAKLGQNLKFFGGGNYNHTFFWESLAPTKQGGGVQPGS      120
Sl-SOD2         -------------AQSKNDVAQISKLGQNLKFFGGGNYNHTFFWESLAPTKLGGGNLPGA      47
                                       : *   ::*  **: **:::* .*:     **

Human MnSOD     KGELLEAIKRDFGSFDKFKEKLTAASVGVQGSGWGWLGFNKERGHLQIAACPNQDPLQG-      172
Ef-SOD2         SGKFGQEVSKTWGSFDNLITDFNTRSAPLQGSGWGWIVYDKNSKALAYTQTFNQDLITE-      173
Tt-SOD2         NSPLTKAIQEKWGSYENFIQIFNGRTAAIQGSGWGWLGYDTVSKSLKMFELGNQDMPE--      98
Ot-SOD2         DSLLTKHINQTWGSYDKFTKNFSDNTGAIQGSGWGWLVYHKGSKCLEFRPSYNQDLITDY      180
Sl-SOD2         DSVLTKHINQTWGSYDKFIANFSAQTASIQGSGWGWLVYHKGSKTLQYRPSYNQDLITDY      107
                .. : : :.. :**::::   :.  :  :*******: :..    *      ***

Human MnSOD     TTGLIPLLGIDVWEHAYYLQYKNVRPDYLKAIWNVINWENVTERYMACKK------    222
Ef-SOD2         KAGLIPLLNVDMWEHAYYLDYKNARPDFLNNIWDVVNWQKIEERFNDATKHHHHHH    229
Tt-SOD2         WNSVIPLLTIDVWEHAYYLDYQNLRPKYLTEIWKVVNWQEVERRYLDAIKA-----    149
Ot-SOD2         QPDLVPLLNIDVWEHAWYLDYKHVKADYLKEIWKVVNWSNVDKRLKEASSQ-----    231
Sl-SOD2         QGDLVPLMNIDVWEHAWYLDYKHVKADYLKEIWKVINWDRVEKRLIAAQKA-----    158
                  .::**: :*:****:**:*:: : .:*. **.*:**..: .*   . .
```

Figure S2. Sequence alignment of Mn SODs from ciliate species and human. Alignment was obtained by ClustalOmega. Residues involved in the coordination of Mn ions are highlighted in magenta.

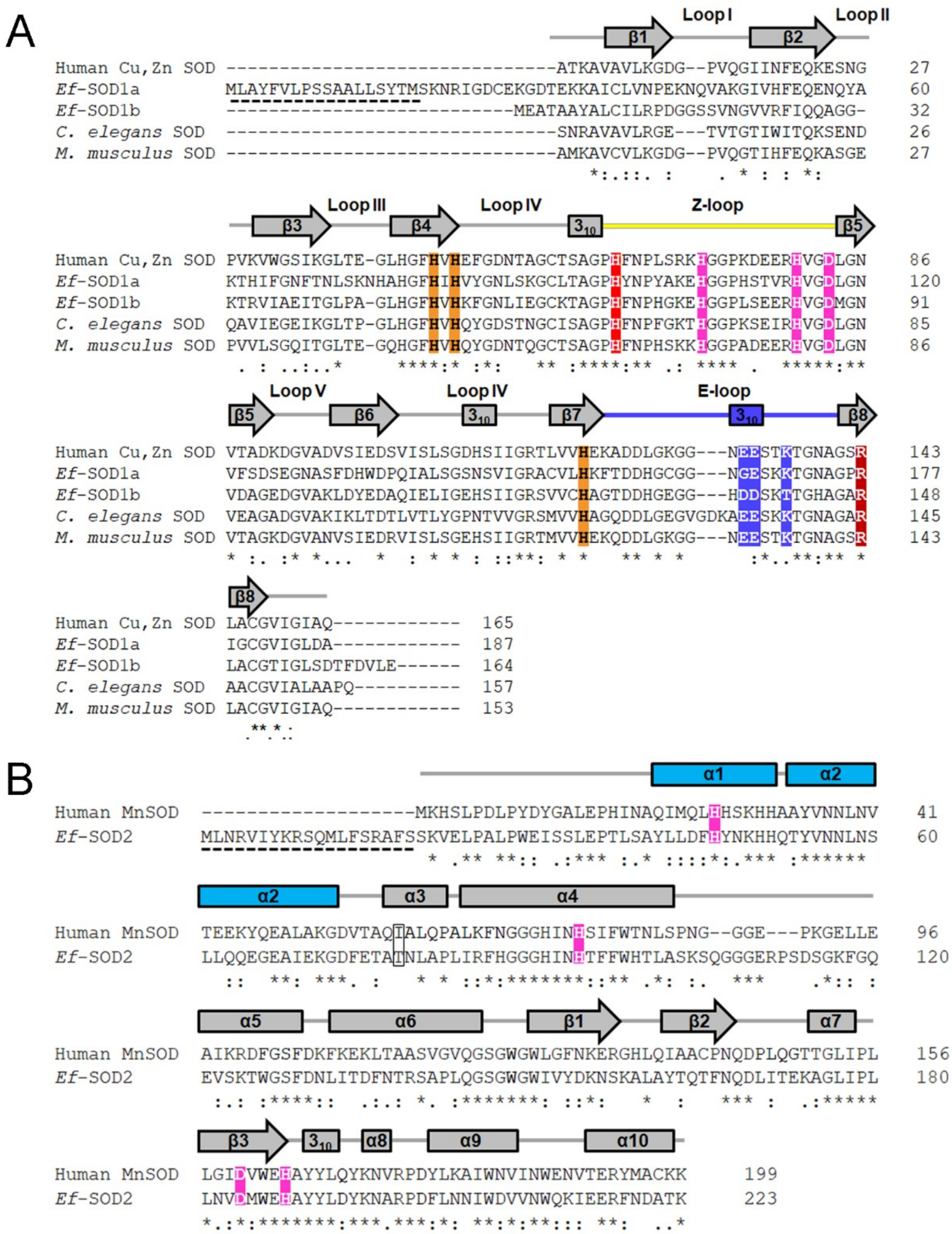

Figure S3. A) Sequence alignment of *Ef*-SOD1s and human Cu,Zn SOD (PDB code: 1NL3), *C. elegans* Cu,Zn SOD1 (PDB: 3KBF) and *Mus musculus* SOD1 (PDB: 3gtt). Color code is the same used in Figure 3A and B. Residues of the electrostatic triad are in blue, Arg residue following the electrostatic triad is in brown. **B)** Sequence alignment of *Ef*-SOD2 and human MnSOD (PDB code: 1VAR). Color code is the same used in Figure 5C. Ile_{58} in human MnSOD and Thr_{78} in *Ef*-SOD2 are boxed. Secondary structure elements extracted from PDB files of human SODs are shown. Signal sequences in *Ef*-SOD1a and *Ef*-SOD2 are underlined by a dotted line. The alignment was obtained by ClustalOmega.

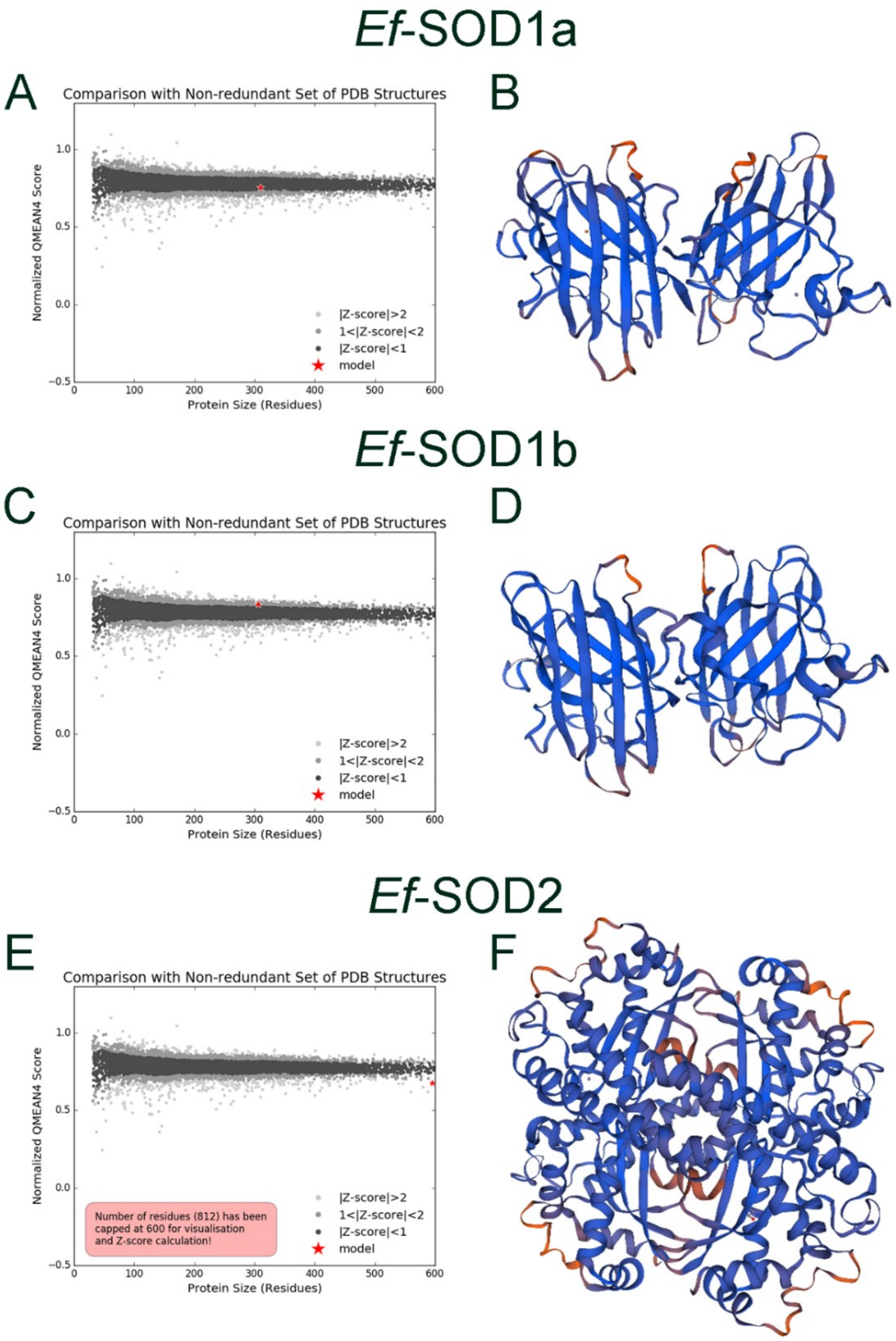

Figure S4. Evaluation of *Ef*-SODs structural models. QMEAN score plot (A, C, E), the normalized QMEAN score is compared with the scores obtained for high resolution crystal structures. Local quality evaluation based on the QMEAN score (B, D, F). Residues with good quality is colored in blue, while the residues poorly modeled are colored in red.

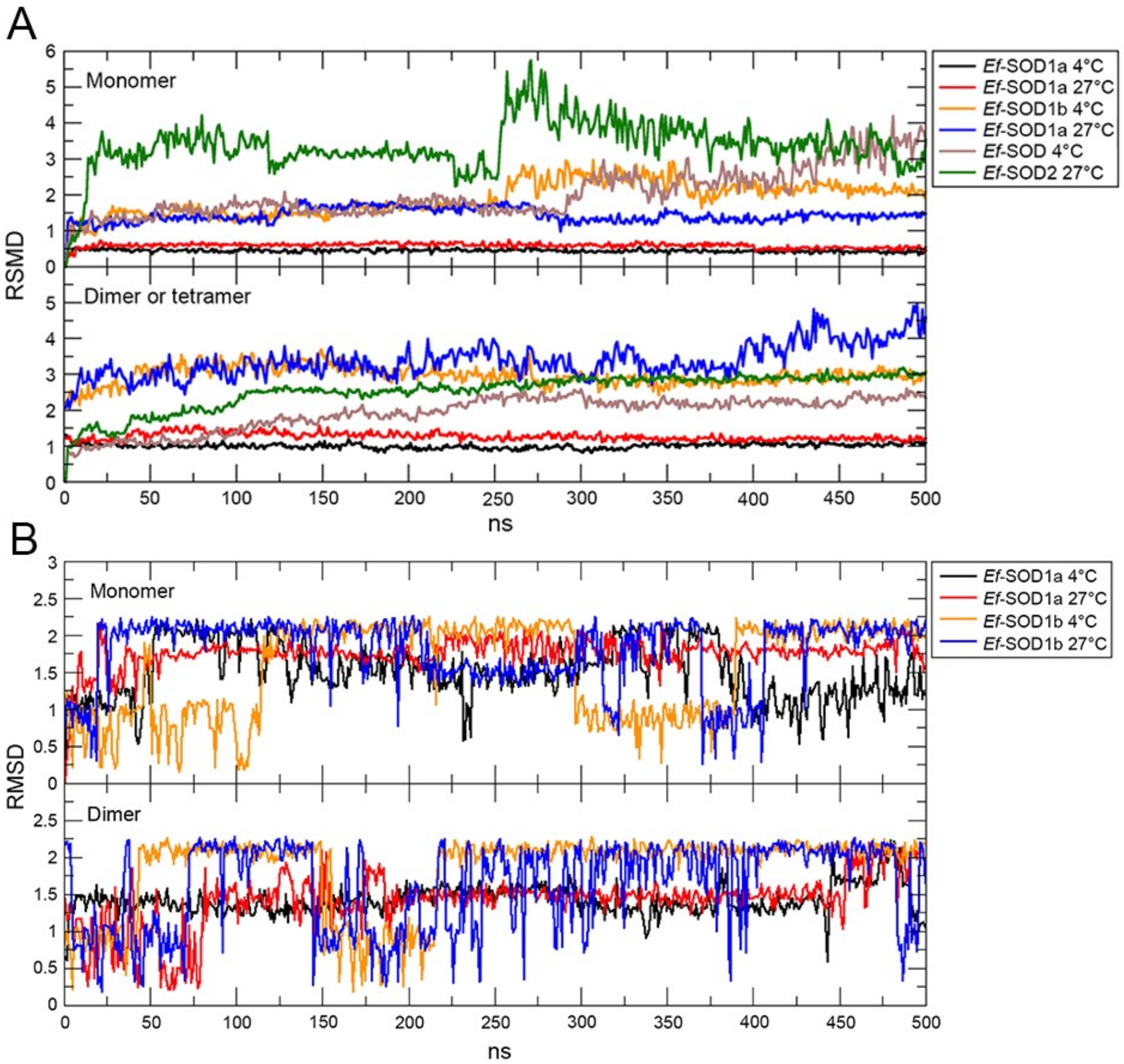

Figure S5. **Molecular dynamic simulations of *Ef*-SODs.** The local flexibility of *Ef*-SODs was evaluated based on the RMSD profile of ion coordination residues (A) and of Arg in position 177 and 148 in *Ef*-SOD1a and *Ef*-SOD1b (B). Simulations were carried out at 4°C and 27°C. Both monomers and oligomers (dimer for *Ef*-SOD1 and tetramer for *Ef*-SOD2) were considered.

Table S1. Sources and accession number of SOD enzymes reported in phylogenetic tree (Figure 1).

Protein ID	Organism	Accession number
Ef-SOD1a	*Euplotes focardii*	KF740481
Ef-SOD1b	*Euplotes focardii*	KF740482
Ef-SOD2	*Euplotes focardii*	MG575644
*Ec*SOD1a	*Euplotes crassus*	contig44280
*Ec*SOD1b	*Euplotes crassus*	contig63897
*Ec*SOD2	*Euplotes crassus*	contig29865
Sl-SOD1	*Stylonychia lemnae*	CDW86167.1
Sl-SOD2	*Stylonychia lemnae*	CDW86249.1
Ot-SOD1a	*Oxytricha trifallax*	EJY88632.1
Ot-SOD1b	*Oxytricha trifallax*	EJY70130.1
Ot-SOD1c	*Oxytricha trifallax*	EJY82908.1
Ot-SOD1d	*Oxytricha trifallax*	EJY71389.1
Ot-SOD2	*Oxytricha trifallax*	EJY66799.1
Tt-SOD1a	*Tetrahymena thermophila*	XP_001007667.2
Tt-SOD1b	*Tetrahymena thermophila*	XP_001033543.1
Tt-SOD1c	*Tetrahymena thermophila*	XP_001032187.1
Tt-SOD2	*Tetrahymena thermophila*	XP_001010506.1
Pt-SOD1a	*Paramecium tetraurelia*	XP_001445360.1
Pt-SOD1b	*Paramecium tetraurelia*	XP_001452078.1
Im-SOD1a	*Ichthyophthirius multifiliis*	XP_004035843.1
Im-SOD1b	*Ichthyophthirius multifiliis*	XP_004036753.1
Dp-SOD1	*Dictyostelium purpureum*	XP_00328385401
Fh-SOD1	*Flavobacterium hibernum*	KIO54302.1
Ns-SOD1	*Nesterenkonia sp AN1*	EXF25878.1
Pa-SOD1	*Planococcus antarcticus*	ANU09845.1
Pp-SOD1	*Pseudocohnilembus persalinus*	KRX07326.1
Ca-SOD2	*Cellulophaga algicola DSM 14237*	ADV48601.1
Ba-SOD2	*Bacillus sp. K2I17*	OWT52390.1
Ps-SOD2	*Pseudomonas sp. KG01*	KMT55131.1
Psa-SOD2	*Pseudoalteromonas sp. ANT 506*	ALN66863.1
Rh-SOD2	*Rhodococcus erythropolis*	ORI29058.1

Table S2. Oligomerization state of *Ef*-SODs. Theoretical molecular mass was calculated from the amino acid sequences using ProtParam, and molecular mass was obtained by SEC-MALS analysis.

	Theoretical molecular mass of monomer (kDa)	Molecular mass (kDa)	Oligomerization state
Ef-SOD1a$^{\Delta}$	19.4	N.A	Dimer*
Ef-SOD1b	17.6	32.8	Dimer
Ef-SOD2$^{\Delta}$	24.5	95.1	Tetramer

Column: Superose12 10/300 GL (GE Healthcare), mobile phase: NaPi 50mM, NaCl 150mM, pH 7.5.

*Obtained by SEC

Table S3: Primers used for quantitative real time PCR and for the deletion of the signal sequences in the deleted variants (Δ). The *NsiI* restriction site is underlined.

Primer name	Forward Primer (5' 3')	Reverse primer (5' 3')
**SSU rDNA**	GTTACGTCCCTGCCCTTTGT	ACCTTGTTACGACTTCTCCTTCC
***Ef*-SOD1a**	TTACAGCTGGACCTCACTATAAC	CACAGCCATGGTCATCTGTAA
***Ef*-SOD1b**	CGGAGGAAAGACCAGAGTTAT	GCCAGCAGTCTTACATCCTT
***Ef*-SOD2**	ATTCCCTCCTTCAGCAAGA	GGAGAGAGACCAAGTGACTCAGG
***Ef*-SOD1a**$^{\Delta}$	AAGAACCGTATCGGTGACTG	ATGCATTATATCTCCTTCTTAAAGTTAAAC
***Ef*-SOD2**$^{\Delta}$	TCATCTAAGGTTGAGCTTCCAG	ATGCATATATCTCCTTCTTAAAGTTAAAC
